# Response to Noise Emitted by Wind Farms in People Living in Nearby Areas

**DOI:** 10.3390/ijerph15081575

**Published:** 2018-07-25

**Authors:** Małgorzata Pawlaczyk-Łuszczyńska, Kamil Zaborowski, Adam Dudarewicz, Małgorzata Zamojska-Daniszewska, Małgorzata Waszkowska

**Affiliations:** 1 Department of Physical Hazards, Nofer Institute of Occupational Medicine, 8, Sw. Teresy Str., 91-348 Lodz, Poland; Kamil.Zaborowski@imp.lodz.pl (K.Z.); Adam.Dudarewicz@imp.lodz.pl (A.D.); Malgorzata.Zamojska@imp.lodz.pl (M.Z.-D.); 2 Department of Work Psychology, Nofer Institute of Occupational Medicine, 8, Sw. Teresy Str., 91-348 Lodz, Poland; Malgorzata.Waszkowska@imp.lodz.pl

**Keywords:** wind turbines, noise, annoyance, health effects

## Abstract

The aim of this study was to evaluate the perception and annoyance of noise from wind turbines in populated areas of Poland. A questionnaire inquiry was carried out among 517 subjects, aged 18–88, living within 204–1726 m from the nearest wind turbine. For areas where respondents lived, A-weighted sound pressure levels (SPLs) were calculated as the sum of the contributions from the wind power plants in the specific area. It has been shown that the wind turbine noise at the calculated A-weighted SPL of 33–50 dB was perceived as annoying or highly annoying by 46% and 28% of respondents, respectively. Moreover, 34% and 18% of them said that they were annoyed or highly annoyed indoors, respectively. The perception of high annoyance was associated with the A-weighted sound pressure level or the distance from the nearest wind turbine, general attitude to wind farms, noise sensitivity and terrain shape (annoyance outdoors) or road-traffic intensity (annoyance indoors). About 48–66% of variance in noise annoyance rating might be explained by the aforesaid factors. It was estimated that at the distance of 1000 m the wind turbine noise might be perceived as highly annoying outdoors by 43% and 2% of people with negative and positive attitude towards wind turbines, respectively. There was no significant association between noise level (or distance) and various health and well-being aspects. However, all variables measuring health and well-being aspects, including stress symptoms, were positively associated with annoyance related to wind turbine noise.

## 1. Introduction

Wind turbines are relatively new sources of community noise and their impact on people’s health and well-being has not been completely explained as yet. Nevertheless, it has been shown that subjects living in the vicinity of wind farms are at the risk of being annoyed by the noise—an adverse impact in itself [1,2,3,4,5]. Noise annoyance in turn could lead to sleep disturbances and psychological distress. No direct effects of wind turbine noise on sleep disturbance or psychological stress have been demonstrated, which means that people who do not hear the sound or do not feel disturbed are not adversely affected [5]. However, the latter effects are still disputable.

Annoyance occurs even when the noise levels do not exceed recommended limits, in particular if multiple sources interact and more than one sensory system is stimulated [6]. Generally, people are more likely to be annoyed when A-weighted sound pressure levels exceed 35−40 dB. The proportion of people perceiving wind turbine noise and annoyed by it increases with increasing noise levels [1,2,3].

Earlier results of laboratory tests implied that none of significant psycho-acoustic parameters explained the observed differentiation in subjective reception of the wind turbine noise annoyance [7]. Instead, the results of subsequent studies [8] demonstrated that analyses of the wind turbine noise annoyance should involve the amplitude of modulation, apart from the sound level. However, it is not certain as yet whether or not at a long distance from wind turbines there is also an amplitude modulation which increases the noise annoyance.

Subjective factors such as having turbines visible from the dwelling, negative opinion about wind turbines in general and/or their visual impact on landscape and self-reported sensitivity to noise increase the probability of being annoyed by the wind turbine noise [1,2], while economic benefits obtained from wind turbines reduce the risk of annoyance [3]. It has been also found that terrain characteristics and urbanization affect the perceived annoyance from the wind turbine noise. In particular, people living in the areas with other background noises are less affected than those from quiet areas [2,5].

It is not quite clear, however, how these factors affect each other and whether or not there is a cause-and-effect relation between them. Furthermore, some feedback may occur between them [9]. Meanwhile the laboratory tests conducted by Maffei et al. [10], using the Immersive Virtual Reality technique, imply that the distance from wind turbines is a factor which affects most the subjective evaluation of noise effects. Along with increasing distance, the perceived sound level, annoyance and stress induced by noise are decreased [10].

On the other hand, according to more recently published findings from Health Canada’s Community Noise and Health Study (CNHS), there is no association between the exposure to the wind turbine noise and the self-reported or objectively measured health endpoints. However, this study results support an association between the increasing wind turbine noise level and an increase in the prevalence of annoyance in relation to various wind turbine features, including noise, perceived indoor vibration during turbines’ operation, visual impacts, shadow flicker, and the aircraft warning lights on top of the turbines [11,12,13,14].

Although wind power has been harnessed as a source of electricity for several decades around the world, its dynamic development in Poland is relatively recent. It is no wonder that the data on reactions to the wind turbine noise in populated areas in Poland are rather sparse.

Recently, Mroczek et al. [15] analyzed the impact of the distance between the place of residence and wind farms on the quality of life in nearby areas, but they did not take into consideration the noise produced by the operation of wind turbines. Their study comprising 1277 people living in rural areas up to 2 km from wind farms showed that a close proximity of wind farms did not result in the deterioration of the quality of life (assessed using the SF-36 General Health Questionnaire). However, the general score of the SF-36 questionnaire obtained by the wind farm area residents was lower than the score achieved by the control group (consisting of 1169 Polish citizens). The most significant differences were observed in domains evaluating general health, physical functioning and mental health [15,16].

The overall aim of this study, which is a continuation of a previous pilot study [17], was to evaluate the perception and annoyance of noise from wind turbines in populated areas in Poland. In particular, the study attempted to:

- Analyze the relationships between the percentage of people being annoyed by the wind turbine noise and noise levels outside their dwellings or the distance from the nearest wind turbine,
- Explore objective (or situational) factors and subjective (or individual) factors affecting the perceived annoyance.

## 2. Material and Methods

A cross-sectional study on the response to the wind turbine noise was carried out, involving people living in the vicinity of ten wind farms located in the northern, central and south-eastern parts of Poland in 2011–2014 (Table 1). Nine areas totaling 223.1 km^2^ were investigated. A questionnaire was applied as the main research tool.

The entire study population comprised 545 subjects aged from 13 to 88 years. They were personally asked to complete questionnaires. Each subject who agreed to participate could be potentially included in the study. However, young people (aged < 18 years) and those living at a distance above 2 km as well as very close (<200 m) to “small” wind turbines were excluded. Thus, the final study group comprised 517 persons, aged 18–88 years, living at a distance of less than 2 km from the nearest wind turbine. For investigated areas, A-weighted sound pressure levels were calculated as the sum of the contributions from the wind power plants in the neighborhood. In addition, for a number of cases the noise conditions outside the dwellings were at random verified by in situ measurements.

### 2.1. Questionnaire Survey

Subjects were personally asked to fill in an anonymous questionnaire developed to enable evaluation of their living conditions, including the prevalence of annoyance due to noise from wind turbines, and the self-assessment of physical health and well-being. To each household at least one copy of the questionnaire was delivered. The response rate was approximately 78%. Almost all subjects (98%) completed the questionnaire themselves, except for some elderly people who were interviewed.

The questionnaire was based on the one previously used in Swedish studies [1,2] and, like the aforesaid questionnaire, it was constructed so as to mask the main intention. The responses to most questions were rated on 5-point verbal rating scales.

The questionnaire consisted of two parts. The first part comprised inquiries about the housing and satisfaction with the living environment, including questions on: (a) occurrence and degree of annoyance experienced outdoors and indoors from various nuisances, including the wind turbine noise, (b) paying attention (being sensitive) to noise, odors and air pollution, landscape littering, (c) general opinion on (attitude towards) the wind turbine and on the visual impact of wind turbines, and (d) different visual and auditory aspects of wind turbines, such as noise, shadows and reflections from rotor blades, during various subjects’ activities (e.g., relaxing, taking walks) and weather conditions.

The second part of the questionnaire was aimed at self-assessment of subjects’ physical health, including their hearing status. It also comprised questions on chronic illnesses (e.g., cardiovascular diseases, hearing impairment, etc.) and general well-being (headache, undue tiredness, pain and stiffness in the back, neck, and shoulders, feeling stressed, irritable), as well as the quality of sleep and normal sleep habits. Statistical analysis of data from the pilot study [17] confirmed a high consistency of questions assessing the response to wind turbines by Cronbach’s α coefficient equal to 0.936.

In addition, the current mental health status of a part (79.5%) of the respondents was assessed using the 12-item Goldberg General Health Questionnaire (GHQ-12) which was adapted for Polish conditions [18,19]. This questionnaire was derived from the main version of the Goldberg General Health Questionnaire. It consists of 12 items describing various symptoms of mental health problems related to two areas, i.e., inability to carry out one’s normal “healthy” functions and appearance of new phenomena of a distressing nature. The subjects are asked to assess the changes in their mood, feelings and behaviors during the recent four weeks using the 4-point response scale (“less than usual”, “no more than usual”, “rather more than usual” and “much more than usual”).

Two methods are used to score the results of GHQ-12. First, responses to each question were coded on the scale from 0 to 3. The total score per subject was obtained by adding the scores for 12 questions. The more mental disorders reported (number and severity of symptoms), the higher the total score of the GHQ-12. In addition, to identify the so-called “cases” (i.e., persons with mental health disorders) the GHQ method was applied for the classification. The answers: “less than usual” and “no more than usual” were coded “0” (lack of symptoms), and the answers “rather more than usual”, “much more than usual” were coded “1” (occurrence of symptoms). In the latter method, the cut-off point between the “non-case” and the “case” for the total score of the questionnaire is 2/3. So, persons with scores 2 or less were classified as “non-cases” (healthy) while persons with scores 3 or more were classified as “cases” [18,19].

### 2.2. Noise Exposure Evaluation

For the areas where the respondents lived, A-weighted sound pressure levels (SPLs) were calculated as the sum of the contributions from the wind turbines in the neighborhood based on the sound propagation model described in ISO 9613-2:1996 [20].

In these calculations, the A-weighted sound power levels of wind turbines specified by manufacturers were used. The arrangements of turbines within each of the farms were obtained from the internet maps (www.geoportal.gov.pl) [21], while the distances between dwellings and turbines were calculated from the GPS data collected in front of the residential premises. Additionally, a correction for the wind velocity distribution was added to the predicted A-weighted SPLs to obtain the day-evening-night noise levels (L_den_) according to 2002/49/EC [22,23].

The calculated SPLs were at random verified by in situ measurements. Relatively quiet areas without too many masking noises (e.g., noises from agricultural machines, hand held and stationary power tools or road-traffic noise) were chosen. Consequently, for a part (38%) of the respondents (*n* = 196), noise levels were measured outside their dwellings at the height of 1.5 and/or 4 m, at the distance of 3 m (or more) from the façade. Measuring points were located next to the respondents’ houses in such a way that the distance from the nearest turbine was shorter than the distance between the turbine and the respondent’s house.

These measurements were carried out according to Polish recommendations on the assessment of environmental noise [24]. However, apart from an equivalent-continuous A-weighted SPL (L_Aeq, T_), other basic noise parameters, such as C- and G-weighted sound pressure levels (L_Ceq,T_ and L_Geq,T_) were measured. In addition, the frequency analysis in 1/3-octave bands from 1.6 Hz to 20 kHz was performed.

Noise measurements were carried out using a type SVAN 958 sound analyzer (SVANTEK, Warsaw, Poland) with a SVANTEK type SV12L preamplifier and a type SV22L microphone equipped with an outdoor kit type SA 277). At each measuring point, at least five noise samples were collected, lasting in total from 5 to 30 min. Particular attention was paid to avoid masking of noises, such as road-traffic noise, dogs barking, etc. However, it was impossible to exclude birds chirping and insects humming.

Measurements were carried out during the daytime, with all or most (>75%) of the turbines installed on the farms working. According to Polish recommendations, the meteorological parameters (i.e., air temperature, air humidity, barometric pressure, wind velocity and direction) were simultaneously monitored at the height of 3.5 m using a weather station (Davis Vantage Vue type 6250, Davis Instruments Corporation, Hayward, CA, USA).

### 2.3. Data Analysis

In the present study, the response to the wind turbine noise was analyzed according to answers to inquiries on: (a) occurrence (“yes” or “no”) and the degree of annoyance experienced outdoors and indoors from this type of noise (“not at all annoying” = 0, “a little annoying” = 1, “rather annoying” = 2, “annoying” = 3, or “extremely annoying” = 4); (b) sensitivity to noise and landscape littering (“definitely no” = 0, “no” = 1, “no opinion” = 2, “yes” = 3, or “definitely yes” = 4); (c) general opinion on (attitude towards) wind turbines and on the visual impact of wind turbines (“very positive” = 0, “positive” = 1, “no opinion” = 2, “negative” = 3,”very negative” = 4), and (d) different auditory aspects of wind turbines during various subjects’ activities (e.g., relaxing, taking walks). The self-assessment of physical health and hearing status (“very poor” = 0, “poor” = 1, “rather poor” = 2, “rather good” = 3, “good” = 4, or “very good” = 5) and prevalence of various aspects of health and well-being (“never”/“almost never” = 0, “several times a year” = 1, “several times a month” = 2, “several times a week” = 3, “everyday/almost every day” = 4) was also analyzed.

When relevant, the data from 5-point (or 6-point) verbal rating scales were dichotomized. In particular, the annoyance scores ≥2 (answers: “rather annoying”, “annoying” and “extremely annoying”) were categorized as “annoying”, while annoyance scores ≥3 (answers “annoying” or “extremely annoying”) as “highly annoying”. On the other hand, the other answers (e.g., “not at all annoying” and “a little annoying”) were categorized as “not annoying” or “not highly annoying”. Similarly, “very poor”, “poor” or “rather poor” self-assessment of physical health or hearing status (scores < 3) were categorized as “negative” while the other (i.e., “rather positive”, “positive” or “very positive”) as positive.

The occurrence of health and well-being aspects (e.g., suffering from headache) at least “a few times per month” (score ≥2) was categorized as “frequent”, while when analyzing the annoyance due to the wind turbine the same description was used in case of answers: “a few times a week” or “everyday”/“almost every day”. Similarly, “very negative” and “negative” attitudes towards the wind turbines (in general and to their visual impact in particular) were categorized as “negative” while the other answers (i.e., “no opinion”, “positive” or “very positive”) as “positive (not negative)”. On the other hand, when analyzing the attention paid to various environmental nuisances, respondents who answered “definitely yes” and “yes” were classified as “sensitive” to noise, landscape littering or air pollution while the others as “not sensitive”.

To analyze the relationships between the distance from the nearest wind turbine and/or levels of wind turbine noise at the dwelling and the percentage of people being annoyed (or highly annoyed) by the noise, the study subjects were classified into subgroups (categories) according to the calculated A-weighted SPL at their dwellings (three noise categories, i.e., up to 40 dB, 40−45 dB and above 45 dB), as well as according to the distance of their dwellings from the nearest wind turbine (three distance categories, i.e., below 400 m, 400−800 m, and above 800 m).

Answers to the questionnaire were presented as the proportions with 95% confidence intervals in the total study group as well as the proportion of respondents in various subgroups. Differences between various pairs of subgroups in proportions of answers were evaluated using the chi-square test.

Relationships between subjective variables and objective variables (i.e., noise and distance categories and noise annoyance rating, noise sensitivity, general attitude to the wind turbine expressed on verbal rating scales, etc.) were analyzed using Spearman’s nonparametric rank correlation coefficient *r*_s_. On the other hand, the association between measured and calculated A-weighted sound pressure levels was assessed using Pearson’s correlation coefficient *r*.

Binary logistic regression was used to study the influence of various objective and individual/subjective factors (e.g., noise level and attitude to the wind turbine) on annoyance related to the wind turbine noise. The same tool was applied to determine the relationships between the percentage of respondents annoyed by the wind turbine noise and various factors, including the calculated A-weighted sound pressure level or distance from the nearest wind turbine. The Nagelkerke pseudo—R^2^ was applied as a measure of explained variance while the correct classification rate (CCR) was considered as a measure of fit of logistic model [25].

The statistical analysis was carried out with an assumed level of significance *α* = 0.05. However, when comparing pairs of various subgroups of respondents or analyzing several relationships at the same time, to avoid the risk of mass significance, *p*-value divided by number (*N*) of possible comparisons or correlations (*p* = 0.05/N) was set as the limit for statistical significance. The statistical analysis employed STATISTICA (version 9.1. StatSoft, Inc.: Tulsa, OK, USA) software package.

## 3. Results

### 3.1. Study Population

The majority of respondents (84.8%) lived in privately owned detached or semi-detached houses in the countryside or in small villages, in rural or suburban areas with diversified terrain shape and road-traffic intensity. Almost all respondents (93.4%) could see one or more wind turbines from their dwelling, backyard or garden. Only a few (6.1%) of them had profits from the wind turbines (Table 2).

The mean age in the study population was 46.7 ± 15.8 years. There were more women than men (58.2% vs. 41.8%, *p* < 0.05). Over half (53.8%) of the respondents were employed, whereas 23.4% of them were pensioners. The majority of subjects had primary (26.3%), vocational (21.8%) or secondary (high school) education (38.5%).

Over half of the subjects were classified as sensitive to noise (68.3%) and landscape littering (63.6%). Of the respondents, 40.3% and 32.3% declared a negative (“very negative” or “negative”) attitude towards wind turbines in general and their visual impact in particular, respectively. Nearly one-third (23.0%) of the subjects assessed their physical health (as “poor” or “very poor”) negatively. Respondents examined using the GHQ-12 obtained the mean score at the level of 12.5 ± 6.1. Furthermore, 35.0% of them were classified as cases according to the GHQ-12 result.

In the majority of cases, there were no significant differences between various noise and distance categories. However, the percentage of subjects negatively assessing wind turbines in general and their impact on landscape in particular, decreased significantly when moving from noise category >45 dB (or 40–45 dB) to <35 dB (*p* < 0.0167). On the other hand, the greater the distance from the nearest wind turbine, the smaller the percentage of subjects with negative opinion on wind turbines (Table 2).

It is worth emphasizing that a majority of subjective (individual) factors (i.e., sensitivity to various environmental nuisances, attitude to wind turbines, physical and mental health status) were correlated to each other (Table 3). In particular, there was a relatively high positive correlation between the attitude to wind turbines in general and attitude to their visual impact in particular (Spearman’s rank correlation coefficient *r*_s_ = 0.757, *p* < 0.0001398) as well as between respondents’ sensitivity to noise and sensitivity to landscape littering (*r*_s_ = 0.674, *p* < 0.00013989).

### 3.2. Noise Exposure Evaluation

**Figure 1:**
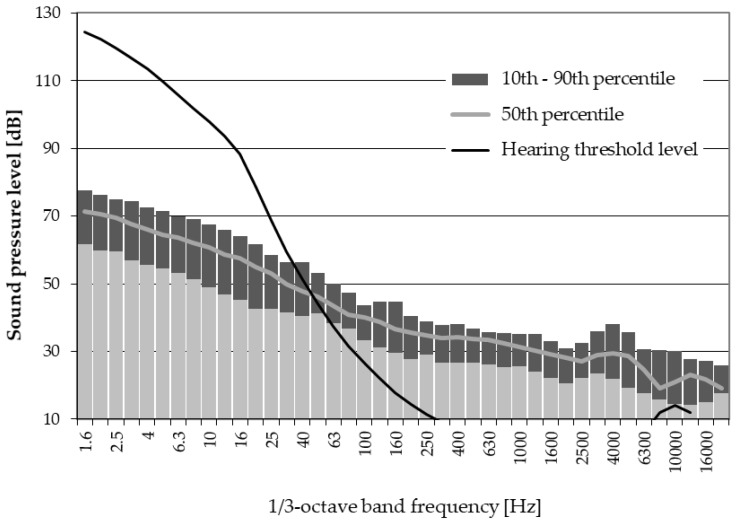
1/3-octave band spectra of noise measured outside respondents’ dwellings together with the hearing threshold level in the infrasonic and audible frequency range [26,27].

The study subjects lived within 204 to 1726 m from the nearest wind turbine. They were exposed to noise at the equivalent-continuous: (a) A-weighted SPLs of 33−50 dB (mean value (M) ± standard deviation (SD): 42.6 ± 3.5 dB, median (Me): 42.6 dB); (b) C-weighted SPLs of 46−70 dB (M ± SD: 57.7 ± 5.7 dB, Me: 58 dB); (c) G-weighted SPLs of 53−90 dB (M ± SD: 72.4 ± 8.6 dB, Me: 72.4 dB). The noise at respondents’ dwellings included infrasonic components but at levels lower than the relevant hearing threshold levels (Figure 1).

It is worth noting that during noise measurements almost all meteorological parameters, excluding air velocity, fulfilled the requirements of the applied measuring method [24]. In particular, atmospheric pressure remained within the range of 974–1028 hPa, relative humidity varied from 25% to 92%, while air velocity ranged from 0 to 20 m/s. The latter parameter did not exceed the limit value (5 m/s) in 79.5% of analyzed cases.

In the areas where respondents lived, the calculated A-weighted SPLs ranged from 33.7 to 49.9 dB, while corresponding exposure metrics L_den_ varied from 35 to 53 dB (Table 4). It should be noted that the mean value of the difference between calculated and measured A-weighted SPLs outside respondents’ dwellings was 0.6 dB (95% CI: −6.5–3.9 dB), while Pearson’s *r* correlation coefficient between these noise levels was 0.31 (*p* = 0.00002).

Over half of the subjects (55.7%) were exposed to noise at the calculated A-weighted sound pressure levels of 40−45 dB, while over two-thirds (66.9%) lived at a distance of 400−800 m from the nearest wind turbine (Table 4).

### 3.3. Questionnaire Survey

#### 3.3.1. Main Results—Assessment of Environmental Conditions

Generally, over half of the respondents paid attention to environmental nuisances at their dwelling places, such as odors and air pollution (65.9%), landscape littering (63.6%) and noise from various sources (68.3%) (see Table 2 for the percentage of the subjects classified as sensitive to the mentioned factors). The most frequently reported nuisances which were noticed outside the dwellings were the wind turbine noise (65.2%), road traffic noise (56.3%), noise from hand held and stationary power tools (47.4%) and noise from agricultural machinery (47.0%).

**Figure 2:**
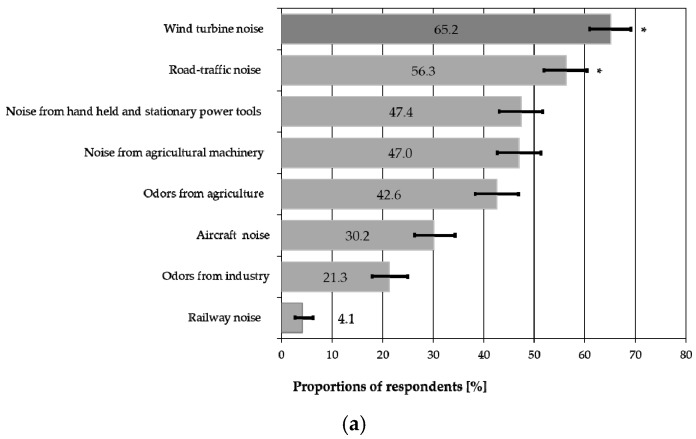

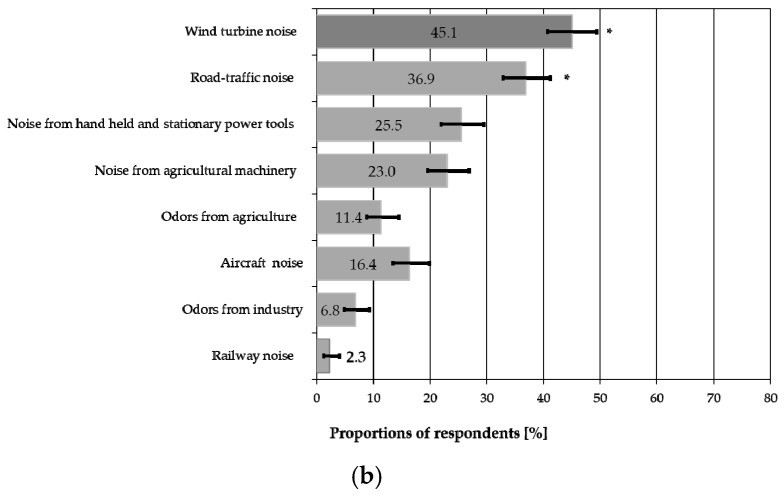
Comparison of proportions (with 95% confidence intervals) of respondents who perceived the wind turbine noise and other environmental nuisances (**a**) outdoors and (**b**) indoors. Cases without a significant difference are denoted (*) (*p* > 0.00625). For other cases, significant differences were found.

There were significant differences between the proportions of subjects perceiving (both outdoors and indoors) the wind turbine noise and other environmental nuisances, excluding the road-traffic noise (Figure 2). The wind turbine noise was perceived by a higher percentage of respondents. Furthermore, it was significantly more frequently perceived as annoying (or highly annoying) than other environmental nuisances, in particular the road-traffic noise (Table 5).

Generally, the wind turbine noise was perceived outdoors by 65.2% of subjects, while 45.1% of them perceived it indoors (Table 6).

**Figure 3:**
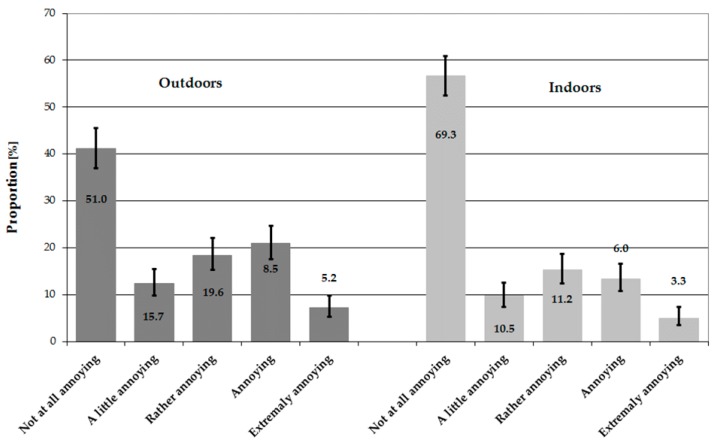
Results of assessment of annoyance due to the wind turbine noise on the 5-point verbal rating scale (outdoors and indoors) in total. Data are presented as proportions with 95% confidence intervals.

Moreover, this type of noise was assessed outdoors as annoying (i.e., as “rather annoying”, “annoying” or “extremely annoying” using a 5-point verbal scale) or highly annoying (i.e., as “annoying” or “extremely annoying”) by 46.4% and 28.0% of respondents, respectively. On the other hand, 33.7% and 18.4% of subjects said that they were annoyed or highly annoyed indoors, respectively. Results of noise annoyance ratings before dichotomization are presented in Figure 3.

**Figure 4:**
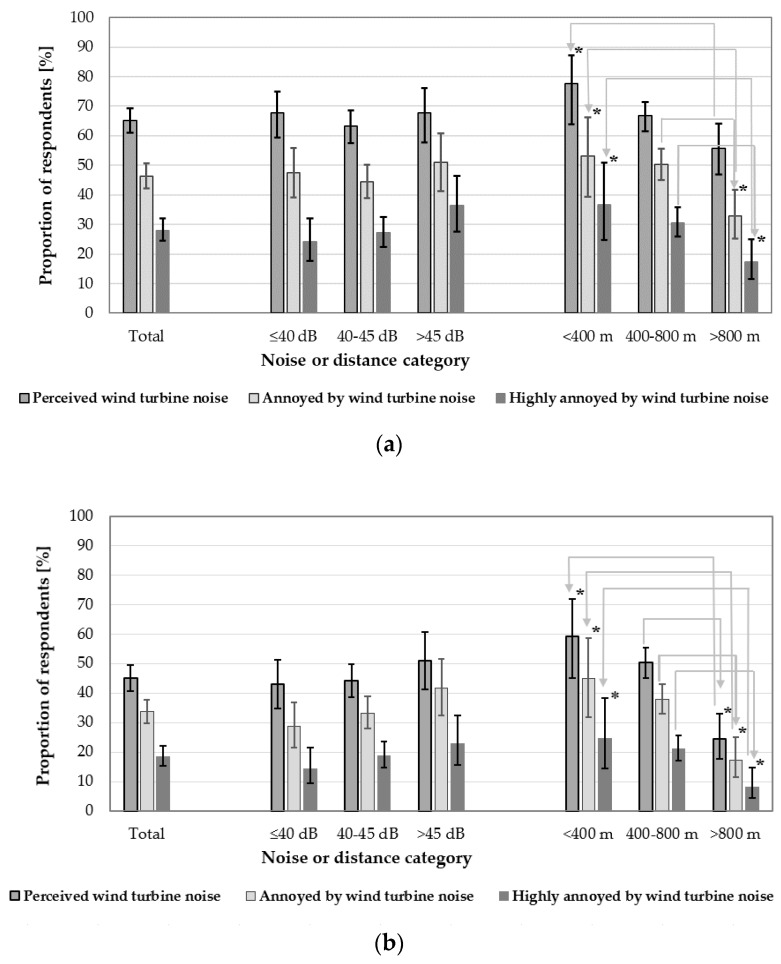
Comparison of proportions (with 95% confidence intervals) of respondents who perceived and were annoyed or highly annoyed by the turbine noise (**a**) outdoors and (**b**) indoors in total and in each of the noise and distance categories. Significant differences between distance categories are marked (*) (*p* < 0.0167).

The proportion of subjects being annoyed (or highly annoyed), both outdoors and indoors, in majority cases decreased significantly with a longer distance. Similar relationships were observed when analyzing the proportions of respondents who perceived the wind turbine noise (both outdoors and indoors) (Table 6 and Figure 4). However, the differences between various distance categories were not significant (*p* > 0.0167).

On the other hand, when analyzing the perception and annoyance of the wind turbine noise (both outdoors and indoors) in various noise categories, no significant differences were noted. The only tendency found was the tendency to increase the percentage of subjects being highly annoyed outdoors or annoyed (and highly annoyed) indoors along with increasing noise levels (Table 6 and Figure 4).

The wind turbine noise was most frequently reported when relaxing outdoors (55.2%, 95% CI: 50.9–59.5%), taking walks (50.9%, 95% CI: 46.5–55.3%) and during quiet outdoor activities (50.5%, 95% CI: 46.1–54.9%) and get-together outdoors such as barbecue (49.6%, 95% CI: 45.2–54.0%). Respondents were also most often annoyed (37.3–40.4%) and highly annoyed (22.8–26.7%) during the mentioned activities.

The percentage of respondents who perceived the wind turbine noise and assessed it as annoying (or highly annoying) during various indoor and outdoor activities decreased significantly along with a longer distance. In particular, the proportions of subjects highly annoyed while relaxing outdoors, taking walks and get-together outdoors decreased significantly from 30.6–34.7% to 11.5–13.1% at a distance below 400 m and above 800 m (*p* < 0.0167), respectively. However, there were no significant differences between noise categories.

It is worth emphasizing that both in case of general questions on evaluation of living conditions and more specific questions concerning wind turbines, the relationships between the distance (or noise) and “high annoyance” were more pronounced than in the case of “annoyance”.

**Figure 5:**
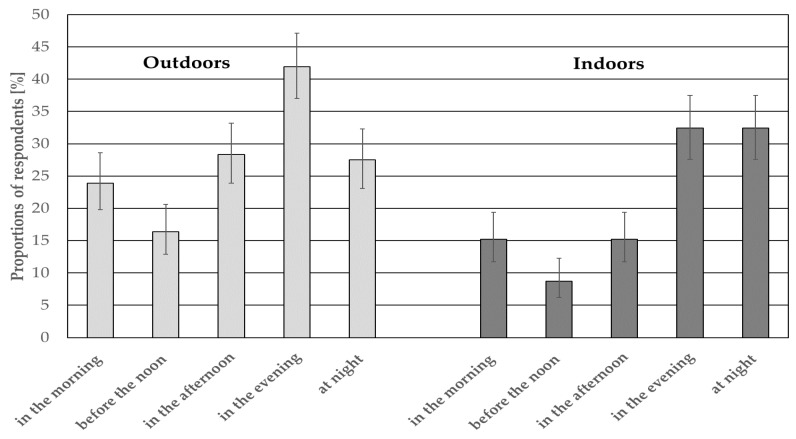
Proportions (with 95% confidence intervals) of respondents being annoyed by the wind turbine noise at different times of the day.

Generally, 40.0% (95% CI: 35.9–44.3%) of the respondents were frequently (“almost every day” or “at least once a week”) disturbed by the wind turbine noise. Proportions of those frequently disturbed by the noise decreased significantly from 44.2% (95% CI: 3.1–49.5%) at a distance of 400–800 m to 26.2% (95% CI: 19.2–34.7%) at a distance above 800 m (*p* < 0.0167), whereas there were no significant differences between various noise categories. Furthermore, outdoors the subjects were most often annoyed by the wind turbine noise in the evening (25.2%, 95% CI: 19.8–31.6%), while indoors—in the evening (16.2%, 95% CI: 11.8–21.8%) and at night (16.2%, 95% CI: 11.8–21.8%) (Figure 5).

About two-thirds of the respondents (64.8%, 95% CI: 60.6–68.8%) indicated rotor blades as the main source of the wind turbine noise, while the noise from the turbine machinery was reported only by 22.1% (95% CI: 18.7–25.8%) of them. Similarly, in all noise and distance categories, higher proportions of respondents perceived noise from the rotor blades than from the machinery. However, the percentage of subjects indicating the rotor blades as the main source of noise was decreased with a higher distance from the nearest wind turbine, while the machinery was more often pointed by respondents living in areas with higher noise levels. Furthermore, a higher percentage of respondents were perceiving noise from the rotor blade, as compared to machinery (50.7 %, 95% CI: 46.4–55.0% vs. 19.3%, 95% CI: 16.2–23.0%, *p* < 0.05) and they often (at least “a few times a week”) found it disturbing (35.85, 95% CI: 31.8–40.0% vs. 15.7%, 95% CI: 12.8–19.1%, *p* < 0.05).

The most frequent verbal descriptors of the wind turbine noise characteristics were “humming” (52.4%, 95% CI: 48.0–56.7%), “very quiet” (19.8%, 95% CI: 16.6–23.6%) and “whistling” (17.5%, 95% CI: 14.4–21.0%). Weather conditions had an impact on the noise perception. Of total respondents, 46.6% (95% CI: 42.4–50.9%) reported that they could hear the noise more clearly than usual when the wind was blowing from the turbine towards their dwelling, while only 8.9% (95% CI: 6.7–11.7%)—when the wind was blowing from the opposite direction. The noise was more clearly heard when a rather strong wind was blowing (51.1%. 95% CI: 46.8–55.3%) and during warm summer nights (28.6%, 95% CI: 24.9–32.7%).

When asked for a general assessment of wind turbines, the respondents most frequently characterized them as “necessary” (38.9%, 95% CI: 34.8–43.2%) and “annoying” (34.2%, 95% CI: 30.3–38.4%). Furthermore, subjects living at a distance <400 m from the nearest wind turbine more often than those living at a distance >800 m described them as “annoying” (42.9%, 95% CI: 30.1–56.7%, vs. 23.0%, 95% CI: 16.4–31.2%, *p* < 0.0167). On the other hand, respondents exposed to a lower sound pressure level (noise category below 40 dB) less frequently than those exposed to a higher noise level (above 45 dB) characterized wind turbines as “necessary” (31.6%, 95% CI: 24.3–39.9%, vs. 50.0%, 95% CI: 40.2–59.8%, *p* < 0.0167).

#### 3.3.2. Self-Assessment of Physical Health and Well-Being

Over one-fifth of the respondents (23.0%) assessed their physical health as “poor” or “very poor” while nearly every fifth or fourth subject frequently (i.e., at least “a few times per month”) reported headache (21.3%), undue tiredness (27.1%), feeling nervous, tense or stressed (28.0%), with pain and stiffness in the back, neck or shoulder (24.2%). Furthermore, of total respondents, 25.1% often had difficulty falling asleep, 19.3% of them complained of insomnia. About half (54.2%) of them often woke up well-rested (badly rested) (Table 7).

Regarding sleep disturbances, 27.3% of the subjects admitted they were disturbed in their sleep by noise from various sources (including the road traffic noise and the wind turbine noise) when sleeping with the window open, while 15.1% of them were thus disturbed when sleeping with closed windows. Moreover, about one-third (33.7%) of the study subjects stated that they were wakened by wind turbines.

Respondents examined using the GHQ-12 obtained the mean score at the level of 12.5 ± 6.1 which was close to the normative result for the reference Polish population (11.17 ± 5.11) [18,19]. Furthermore, the prevalence of “cases” (35%) in the study population was comparable to that observed in the healthy working Polish population (27%) [18].

**Figure 6:**
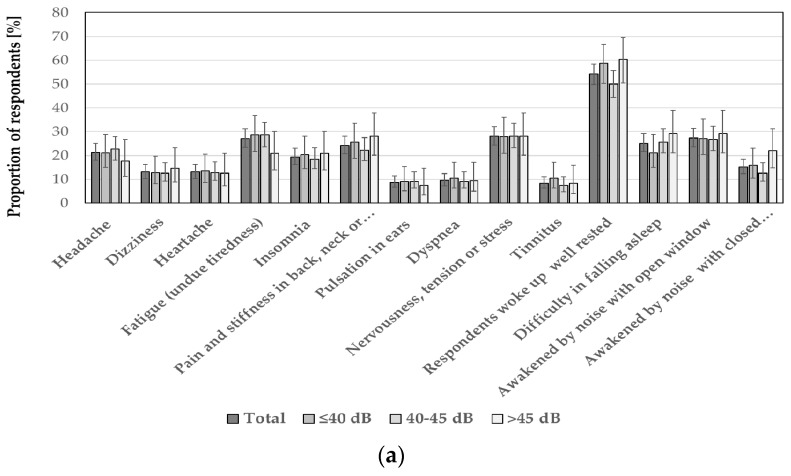

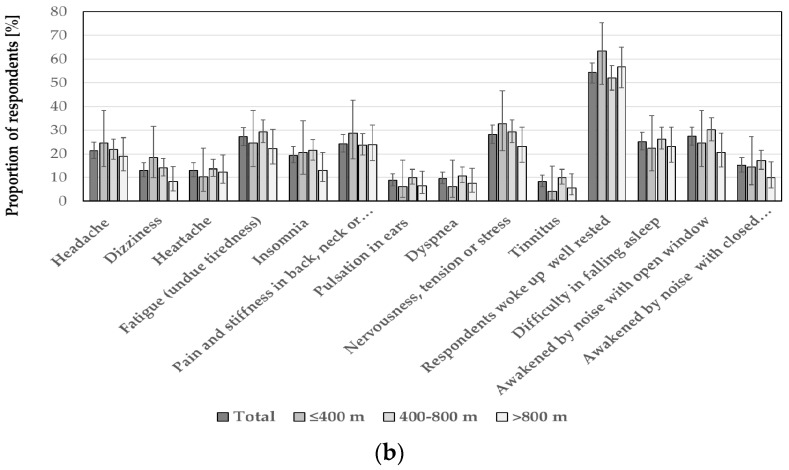
The prevalence of various health and well-being aspects in total and in each of the: (**a**) noise and (**b**) distance categories. Data are presented as proportions with 95% confidence intervals. There were no significant differences between various noise and distance categories (*p* > 0.00167).

As to various health and well-being aspects, there were no significant differences between noise or distance categories (Figure 6). However, the people being annoyed or highly annoyed by the wind turbine noise more frequently than those not annoyed reported various health symptoms and assessed their physical health negatively. Furthermore, a higher percentage of the so-called “cases” according to the GHQ-12 score were recognized among annoyed (or highly annoyed) subjects (Table 7).

### 3.4. Factors Affecting Perception of Annoyance and Self-Reported Health

According to earlier studies, including our pilot study [17], apart from the noise level (or the distance from the nearest wind turbine) many objective and subjective factors such as respondents’ general attitude to wind turbines, sensitivity to landscape littering, financial benefits etc. were found to have a significant impact on the perceived annoyance [1,2,3,5,17]. On the other hand, the prevalence of the health symptoms can vary with age and between males and females. Thus, in order to analyze the influence of various variables (including the noise level and distance) on annoyance and different health aspects related to the wind turbine noise, like in the earlier study [1,28], the binary logistic regression was applied with the logistic model expressed as follows:

(1) $$p=\frac{e^{{(b}_{0}+b_{1}x_{1}+b_{2}x_{2}+\ldots +b_{n}x_{n})}}{1+e^{{}^{{(b}_{0}+b_{1}x_{1}+b_{2}x_{2}+\ldots +b_{n}x_{n})}}}\;$$

where: p is the probability of outcome (i.e., being highly annoyed or occurrence of health symptom), x_1_–x_n_—are the explanatory variables included in the model (e.g., calculated A-weighted sound pressure level), b_0_, b_1_, … b_n_—are the regression coefficients, i.e., the logarithmic values of the odds ratio for the unit change in the respective variables.

It is worth noting that this method allows adjustments for known confounders by entering them into regression analyses (e.g., variables age and sex when analyzing the prevalence of health symptoms). An odds ratio (with 95% confidence interval) above 1.00 indicates a positive correlation between the dependent variable (e.g., health symptoms) and explanatory variable (e.g., noise level or annoyance), while a value below 1.00 indicates a negative correlation between the dependent variable (e.g., annoyance) and explanatory variable (distance or general attitude towards wind turbines).

#### 3.4.1. Annoyance Related to Wind Turbine Noise

To analyze the factors affecting annoyance perception, various models were created containing the calculated A-weighted sound pressure level (noise level) at the respondent’s dwelling or distance from the nearest turbine as the main potential explanatory variable. Other analyzed explanatory variables were: age (in years), gender (“female” = 0, “male” = 1), education (1–5), sensitivity to noise or landscape littering (0–4), general attitude to wind farms (0–4), terrain shape (“flat” = 0, “hilly or mountainous” = 1), road-traffic intensity (“increased intensity” = 0, “no road-traffic or very low-intensity traffic” = 1), time since the start-up of the wind farm (1–11 years), power (0.132–2.5 MW) and height (30–103 m) of the nearest wind turbine, economic benefits from the wind farm and having wind turbines visible from the dwelling (“no” = 0, “yes” = 1). Furthermore, due to more pronounced association between noise (or distance) categories and high annoyance, in the following part only the probability of being highly annoyed by the wind turbine noise was analyzed.

In the first step, eight models describing the high annoyance perception (outdoors and indoors) were analyzed (Table 8 and Table 9). According to these basic models, the probability of being highly annoyed outdoors was significantly associated with the sound pressure level (or distance), attitude towards wind turbines, sensitivity to noise as well as terrain shape. On the other hand, the annoyance perception indoors was correlated with the noise level (or distance), age, attitude towards wind turbines, sensitivity to noise and traffic intensity. Neither gender nor education had a significant impact on the noise annoyance rating. Likewise, no significant relationships were observed in case of the remaining objective or situational factors.

In the second step, various models (describing the probability of being highly annoyed by the wind turbine outdoors and indoors) were created taking into account the aforesaid independent variables, including those containing only the sound pressure level or distance as explanatory variables (Table 10 and Table 11). It is worth emphasizing that majority regression coefficients in these models, excluding some regression coefficients for the sound pressure level and distance, reached statistical significance (see models numbered 13 and 14 in Table 10 and models 1, 3 and 6 in Table 11).

When analyzing the probability of being highly annoyed outdoors, the highest values of pseudo-R^2^ equal to approximately 0.66 were obtained for models No. 1 and 8 (see Table 10), indicating that the sound pressure level (or distance), terrain shape, sensitivity to noise and general attitude to wind turbines explained 66% of variance in annoyance assessment. Similar results, i.e., pseudo-R^2^ of 0.60–0.65, were obtained for models No. 2–4 and 8–11, in particular those containing only noise level (or distance) and general attitude to wind turbines as explanatory variables (model No. 4). On the other hand, the distance itself explained only about 1% of the variance in perceived annoyance (see model No. 7 in Table 10).

Similar relationships were obtained when studying the noise annoyance perception indoors (Table 13). However, in this case, instead of the terrain shape, the road-traffic intensity was taken into consideration. Furthermore, the highest value of explained variance in annoyance equal to approximately 48% was obtained for the model containing the attitude to wind turbine, sensitivity to noise, road-traffic intensity and noise level as explanatory variables. In turn, distance (or sound pressure level) itself explained only about 2% of the variance in perceived high annoyance indoors (see models no. 8 and 14 in Table 11).

The odds ratio of being annoyed (or highly annoyed) by the wind turbine noise (both outdoors and indoors) increased with an increasing noise level (OR > 1.00), higher sensitivity to noise (OR > 1.00) and more negative attitude towards wind turbines, and decreased with an increasing distance from the nearest wind turbine (OR < 1.00), provided that the regression coefficients reached a statistical significance. Likewise, hilly or mountainous (vs. flat) terrain and no or low-intensity (vs. increased intensity) road-traffic increased the risk of being annoyed outdoors and indoors, respectively.

#### 3.4.2. Exposure-Response Relationships

Results of the logistic regression analysis were also a basis for determination of exposure-response relationships expressed as probability (percentage) of subjects being highly annoyed outdoors or indoors versus noise level (or distance) and other variables.

**Figure 7:**
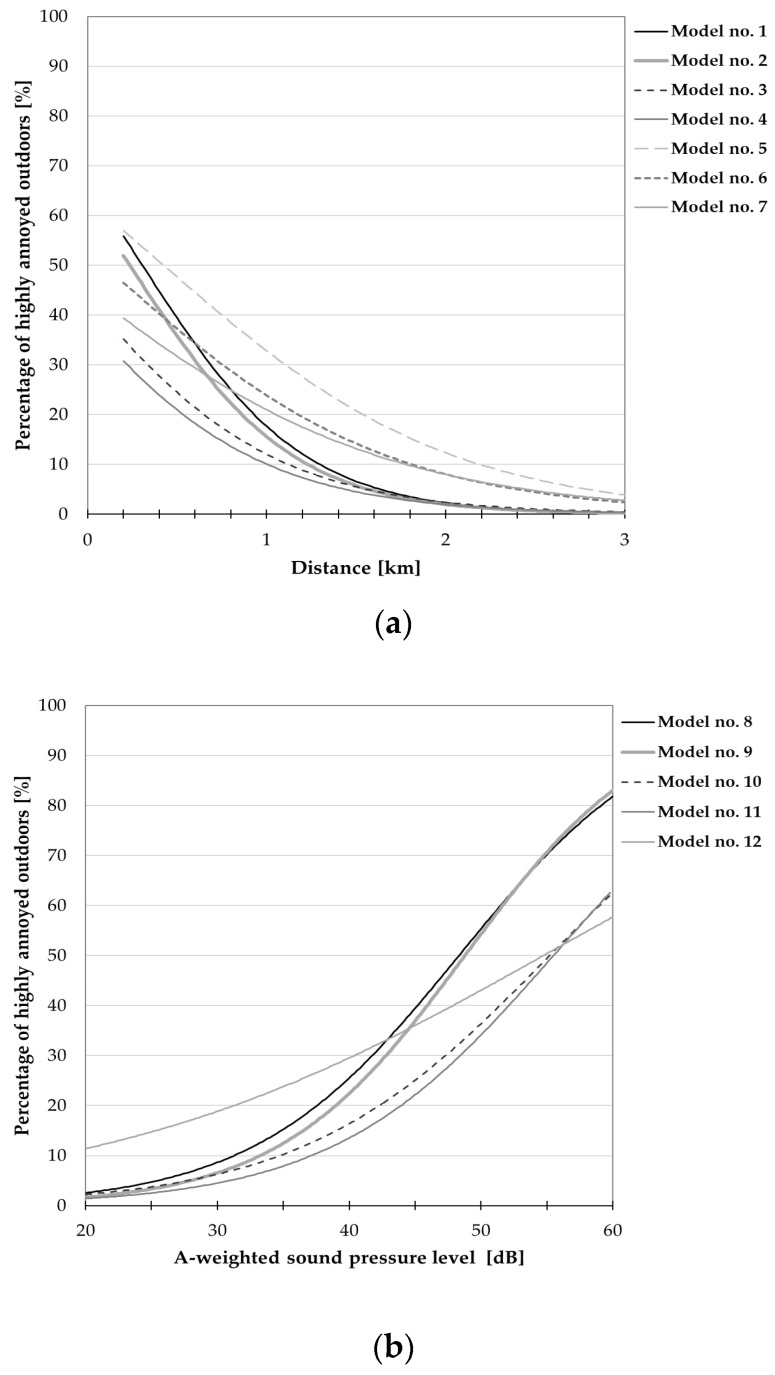
Relationships between the percentage of respondents being highly annoyed outdoors and (**a**) noise level or (**b**) distance from the nearest wind turbine. The curves correspond only to those models from Table 12 for which all regression coefficients reached a statistical significance; they were drawn, where applicable, with explanatory variables of “terrain shape” (=1), “sensitivity to noise” (=3) and “attitude to wind farms” (=2) equal to median values in the study group.

**Figure 8:**
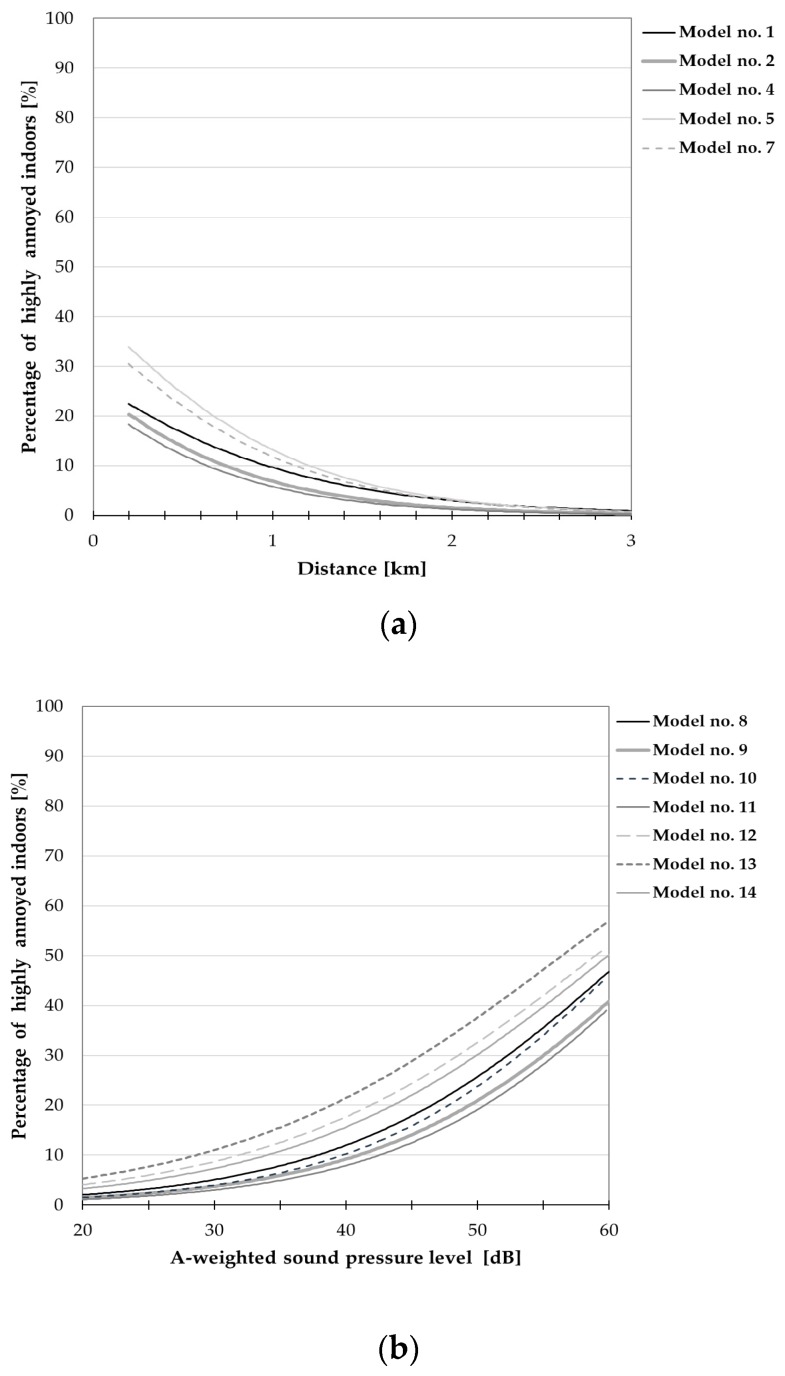
Relationships between the percentage of respondents being highly annoyed indoors and (**a**) noise level or (**b**) distance from the nearest wind turbine. The curves correspond only to those models from Table 13 for which all regression coefficients reached a statistical significance; they were drawn, where applicable, with explanatory variables of “road-traffic intensity” (=1), “sensitivity to noise” (=3) and “attitude to wind farms” (=2) equal to median values in the study group.

**Figure 9:**
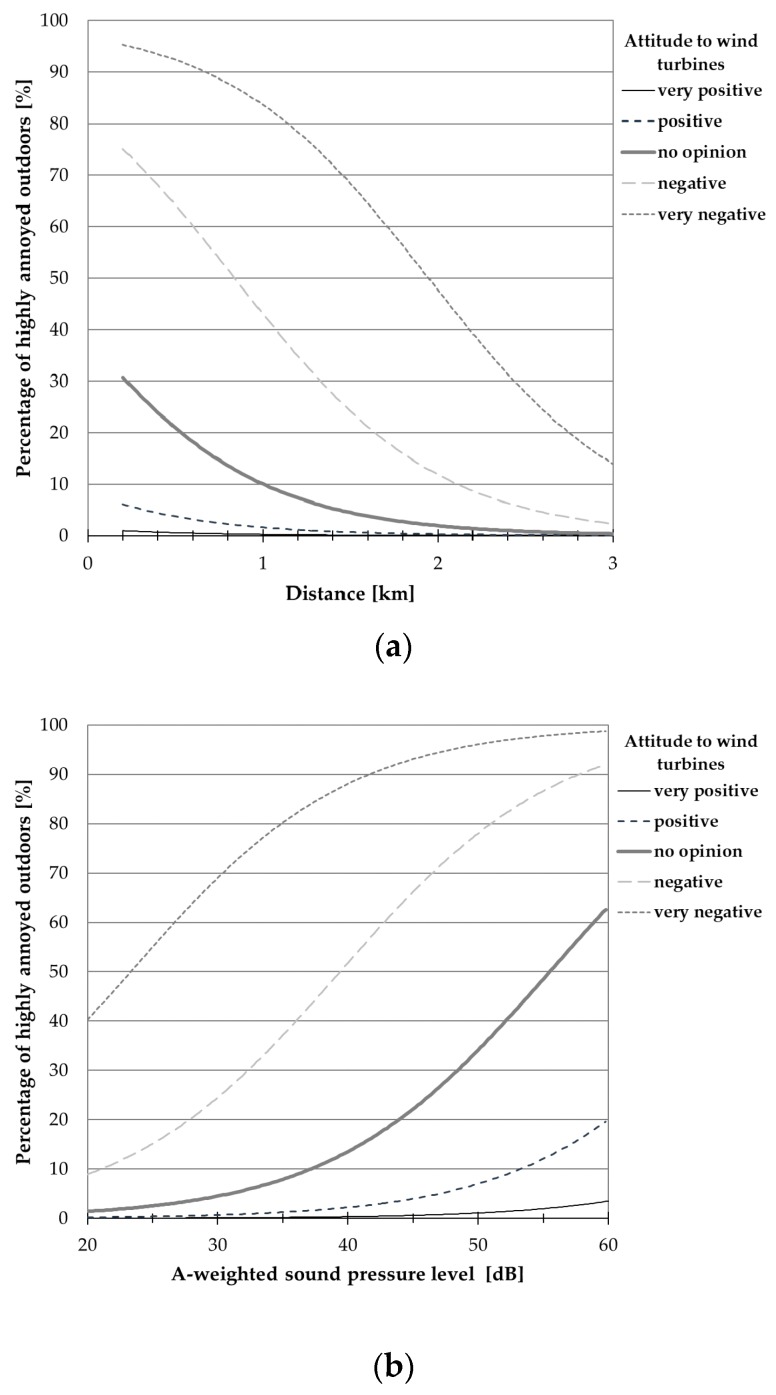
Percentage of respondents being highly annoyed outdoors by the wind turbine noise as a function of attitude to wind farms and (**a**) noise level or (**b**) distance from the nearest wind turbine.

**Figure 10:**
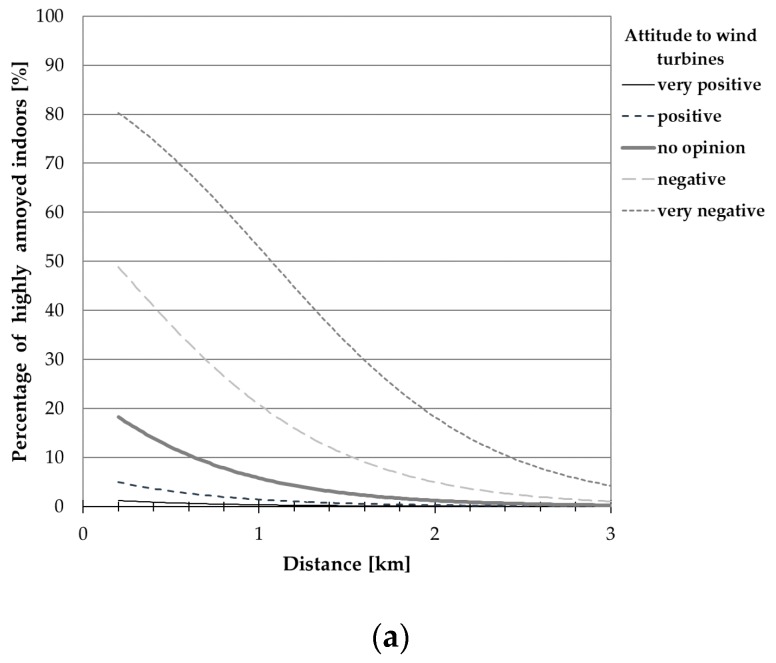

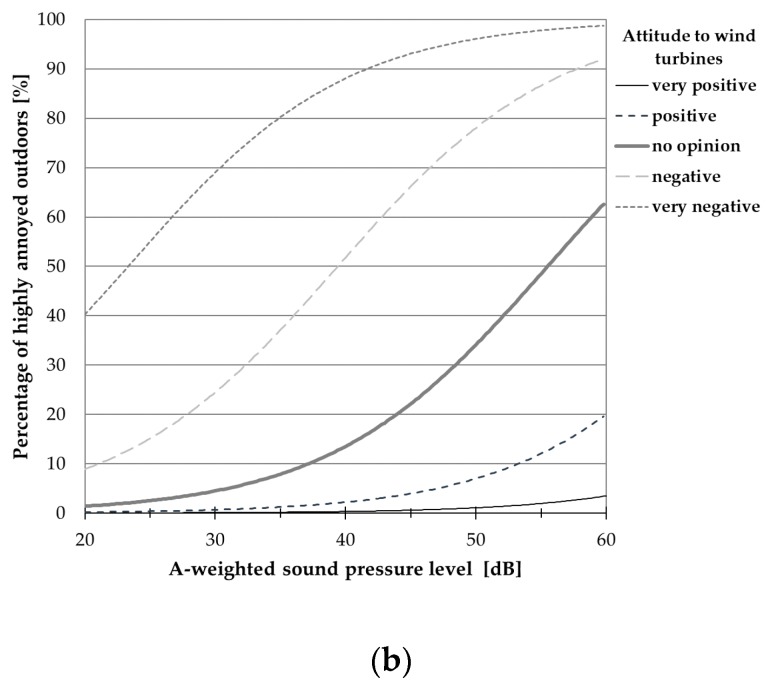
Percentage of respondents being highly annoyed outdoors by the wind turbine noise as a function of attitude to wind farms and (**a**) noise level or (**b**) distance from the nearest wind turbine.

Table 12 and Table 13 present the estimates of regression coefficient β_i_ (with estimated standard errors) for the aforesaid models, while Figure 7, Figure 8, Figure 9 and Figure 10 show the exposure-response curves corresponding to models for which all regression coefficients reached a statistical significance. These curves were drawn, where applicable, with explanatory variables such as the terrain shape (or road-traffic intensity), sensitivity to noise and attitude to wind turbines equal to median values in this study population.

On the other hand, Figure 9 and Figure 10 present exposure-response curves which take into consideration, apart from the noise level (or distance), a general opinion about wind turbines. According to these curves, namely those corresponding to model no. 4 in Table 12 (Figure 9a) at the distance of 1000 m, the wind turbine noise might be perceived as highly annoying outdoors by approximately 2% and 43% of people with positive and negative attitudes towards wind farms, respectively. At the same distance, up to 21% of the subjects (with negative attitude to wind farms) might be highly annoyed by the wind turbine noise indoors (see Figure 10a, model No. 4 in Table 13). On the other hand, in areas where the A-weighted sound pressure level exceeds 40 dB, above 2 and 52% of people with a positive and negative attitude to wind farms, respectively, might be highly annoyed outdoors (see Figure 9b, model no. 11 in Table 12). At such noise levels, above 27% of subjects with a negative opinion about wind farms might be annoyed indoors (see Figure 10b, model no. 11 in Table 13). However, regardless of the noise level or distance from the nearest wind turbine, a much lower percentage of the subjects who are indifferent to wind farms might perceive the wind turbine noise as highly annoying (Figure 9 and Figure 10).

#### 3.4.3. Factors Affecting Perception of Self-Reported Health

As mentioned above, the relationships between noise levels (or distance) and self-reported health and well-being were also tested with the binary logistic regression. Since the prevalence of the health symptoms can vary with age and gender, thus these two explanatory variables were taken into consideration. Furthermore, the associations between high annoyance, both outdoors and indoors, were analyzed. In the latter case, apart from age and gender, noise level (or distance) was included into models as a confounder.

As we can see in Table 14, neither the noise level nor the distance from the nearest wind turbine had an impact on various health and well-being aspects. Furthermore, binary logistic regression revealed that almost all variables measuring various health and well-being aspects were positively associated with a high annoyance related to the wind turbine noise both outdoors and indoors. A negative relationship was only observed in case of a self-reported quality of sleep.

## 4. Discussion

One of the most widely investigated responses to environmental noise is an annoyance. Wind turbines are relatively new sources of community noise and their influence on health and well-being of people living nearby has not been completely determined as yet.

For years, most epidemiological evidence concerning the impact of the modern wind turbine noise on people’s health and well-being has been coming mainly from three cross-sectional studies carried out in Sweden and the Netherlands between 2000 and 2007 [1,2,3,5]. Later, cross-sectional studies on human responses to wind turbine noise have been conducted also in other countries, including New Zealand [29], USA [30,31], China [32], Canada [11,12,13,33] and Poland [15,16,17,18]. A questionnaire was applied as the main research tool in almost all of them, excluding the recent Community Noise and Health Study (CNHS) in Canada which assessed responses to the wind turbine noise using both self-reported and objective measures (e.g., measures of blood pressure and heart rate) [13]. It is worth noting that a number of the aforesaid investigations used questionnaires which were designed according to the one previously used in Swedish studies [31,32].

Noise exposures in areas where respondents lived were mainly estimated using the A-weighted sound pressure levels which were calculated from sound power levels of all wind turbines nearby. These calculations were usually supplemented (or verified) by in situ noise measurements [1,2,3,5,32]. However, Magari et al. [31] based their study on short-term outdoor and indoor sound pressure level measurements at respondents’ dwellings. On the other hand, in the Canadian CNHS, both the A- and C-weighted sound pressure levels were calculated for determination of exposure response relationships [11].

In this study, which is a continuation of the previous pilot study, similarly to the aforesaid Scandinavian cross-sectional investigations, the basis of analyses were the results of questionnaire surveys and calculated A-weighted SPLs for areas where respondents lived, which were verified at random by in situ short-term sound pressure level measurements. However, apart from the basic questionnaire (aimed at evaluation of annoyance due to the wind turbine noise and self-assessment of physical health and wellbeing) the respondents were additionally asked to complete the 12-item Goldberg General Health Questionnaire to assess their mental health status. It is worth emphasizing that this questionnaire was used earlier by Bakker et al. [5] to assess the non-specific psychological distress.

In this study, the calculated A-weighted sound pressure level (determined according to the sound propagation model described in ISO 9613-2:1996) ranged from 33 to 50 dB, while the measured A-weighted equivalent-continuous SPL remained within the range from 34 to 50 dB.

The wind turbine noise prevailing at respondents’ dwellings included infrasonic components, but at levels lower than the hearing threshold levels (see Figure 1). The G-weighted equivalent-continuous sound pressure level ranged from 53 to 90 dB within 204–1726 m from the nearest turbine. These results were not surprising since all wind turbines in this study were upwind devices [34].

Earlier, O’Neal et al. [35] performed noise surveys outside and within nearby residences of wind turbines from two different manufacturers and they also found that the measured (at distances of 305 m and 457 m) sound pressure levels (in 1/3-octave bands) in the infrasonic range were lower than hearing threshold levels. More recently, results similar to our results have been obtained by Song et al. [32] who analyzed noise impacts induced by 2 MW wind turbines in China. Although their sound pressure levels in 1/3-octave bands were higher due to a larger single-machine capacity of wind turbines and a shorter distance between measuring points and the nearest wind turbines, the range of audible frequencies was almost the same, and infrasonic components were also lower than relevant hearing threshold levels [32].

To evaluate the annoyance due to noise from wind turbines, participants of this study completed a questionnaire, which was based on the one previously used in Swedish and Dutch studies [1,2,3]. Furthermore, to reduce a self-reporting survey bias, like that inherent to the aforesaid questionnaire, it included also questions about several potential environmental stressors and did not allow respondents to realize that the focus of the study was on the wind turbine noise. It is worth noting that there was a high correspondence between the responses to the general questions of noise from the wind turbine at the beginning of the Swedish questionnaire and the more specific questions later (expressed by Cronbach’ s α coefficient equal to 0.885) [1]. Statistical analysis of data from the pilot study also confirmed a high internal consistency of different questions evaluating the response to wind turbines (*α *= 0.93) [17].

Generally, earlier cross-sectional studies showed that the proportion of people being annoyed by the wind turbine noise increased along with increasing noise levels [1,2,3,5] or reduced proximity to wind turbines [29]. It has also been found that subjects were more likely to be annoyed when calculated A-weighted sound pressure levels exceeded 35–40 dB [2,3]. Furthermore, the wind turbine noise was found to be more annoying than transportation noise or industrial noise at comparable levels, possibly due to specific sound properties such as a “swishing” quality, temporal variability, and lack of the night time abatement [3]. Some personal factors (such as having turbines visible from the dwelling, negative opinion about wind turbines in general and/or their visual impact on landscape and self-reported sensitivity to noise) appeared to increase the odds of being annoyed by the wind turbine noise [1,2].

On the other hand, especially in the Dutch studies, the risk of annoyance was considerably lower in the subjects obtaining economic benefits from wind turbines [3]. Moreover, according to the aforesaid studies the perception and annoyance were associated with the terrain and urbanization, i.e., a rural area increased the risk of perception and annoyance in comparison with a suburban area, in a rural setting a diversified (hilly or rocky) terrain increased the risk compared with a flat terrain [2]. In turn, according to more recently published results of the Canadian study (CNHS), variables associated with the wind turbine noise annoyance comprised, but were not limited to, other wind turbine-related annoyances (e.g., shadow flickers), personal benefits, noise sensitivity, physical safety concerns, property ownership and province of Canada [12].

Our pilot study included only respondents living in the countryside or in small villages located in the rather flat and mainly agricultural terrain with low traffic intensity. However, the final study population included inhabitants of rural and suburban areas (near small towns) with diversified terrain shape and road-traffic intensity. Thus, we could analyze the impact of terrain shape and road-traffic intensity on annoyance related to the wind turbine noise.

To explore the prevalence of annoyance related to the wind turbine noise, when relevant answers to questionnaire on 5- or 6-point verbal rating scales were dichotomized. In particular, likewise in case of transportation and industrial noise, where the cutoff at 50 and 72 on a 0–100 rating scale was related to terms “annoyed” and “highly annoyed”, respectively [36], in this study annoyance ratings ≥2 and ≥3 (on 0–4 scale) were categorized as “annoying” and “highly annoying”, respectively.

It appeared that the wind turbine noise at calculated A-weighted sound pressure levels of 34–50 dB was perceived outdoors as annoying (i.e., as “rather annoying”, “annoying” or “extremely annoying”) or highly annoying (i.e., as “annoying” or “extremely annoying”) by 46.4 and 28.0% of respondents, respectively. On the other hand, 33.7% and 18.4% of them said that they were annoyed or highly annoyed indoors, respectively.

Recently, based on the available data from Swedish and Dutch cross-sectional studies [1,2,3], the exposure-response relationships between the exposure metric L_den_ (annual day-evening-night noise level according to 2002/49/EC [22]) and self-reported annoyance indoors as well as outdoors of the dwellings due to the wind turbine noise were determined using the method previously applied to derive the exposure-response relationships for transportation and industrial noise [37]. To obtain the exposure metrics L_den_, a correction of +4.7 dB, calculated by van den Berg [23], was added by Janssen et al. [37] to the predicted A-weighted SPLs. Finally, four exposure-response relationships showing the percentages of annoyed and highly-annoyed residents by the wind turbine noise at given L_den_, were determined. Generally, in comparison to other sources of environmental noise, annoyance due to the wind turbine noise was found at relatively low noise exposure levels.

To compare the percentages of subjects being annoyed (or highly annoyed) by the wind turbine noise observed in this study with the predictions of the aforesaid exposure-response relationships, the calculated A-weighted sound pressure levels were also corrected to obtain the exposure metrics L_den_ (Table 4). Furthermore, the study population was divided into 10 equinumerous categories due to the L_den_ level and proportions of subjects being annoyed and highly annoyed were determined.

**Figure 11:**
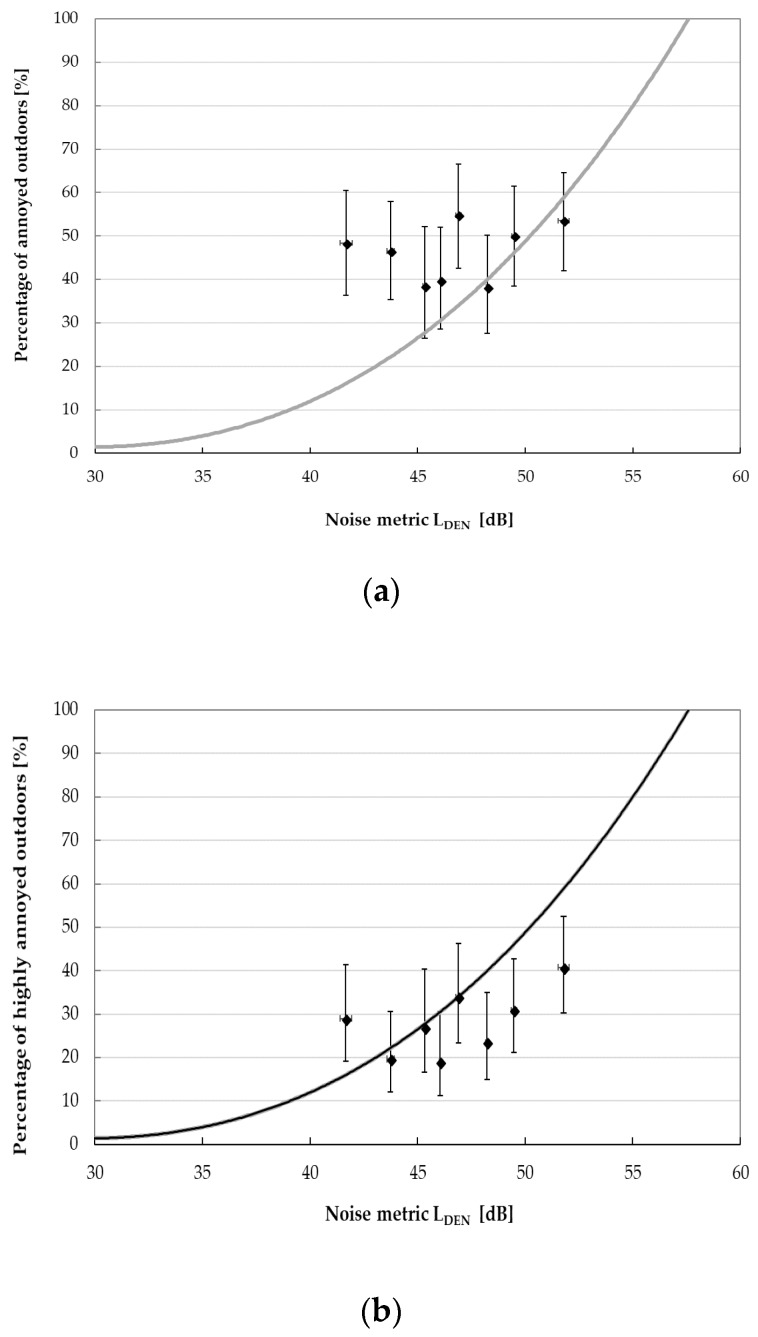
Comparison of observed proportions (with 95% confidence levels) of respondents being (**a**) annoyed and (**b**) highly annoyed outdoors by the wind turbine noise to exposure-response relationships for wind turbine annoyance outdoors proposed by Janssen et al. Dots with whiskers represent survey data with 95% confidence intervals.

**Figure 12:**
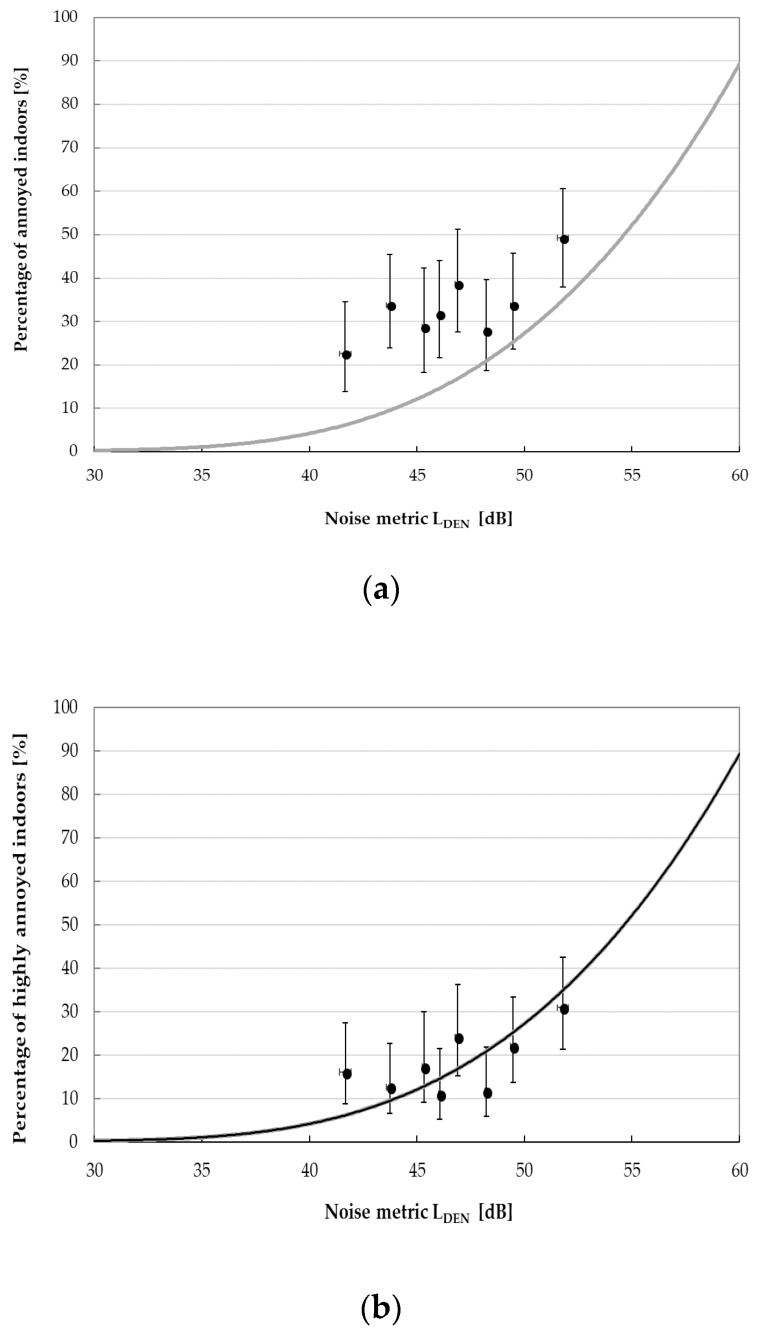
Comparison of observed proportions (with 95% confidence levels) of respondents being (**a**) annoyed and (**b**) highly annoyed indoors by the wind turbine noise to exposure-response relationships for wind turbine annoyance outdoors proposed by Janssen et al. Dots with whiskers represent survey data with 95% confidence intervals.

The percentages—observed in this study—of respondents being highly annoyed, both outdoors and indoors, by wind turbines noise at given L_den_ levels fitted quite well the proposed exposure-response curves (Figure 11b and Figure 12b). However, when analyzing the percentage of subjects being annoyed (outdoors or indoors), a good agreement with the aforesaid predictions was noted for the L_den_ levels above 45 dB (Figure 11a and Figure 12a).

As mentioned above, a general conclusion from other previous studies was that annoyance increased with increasing wind turbine noise levels [1,2,3,5,11] or reduced proximity to wind turbines [29]. For example, in a recent Canadian study the increase in high annoyance was clearly evident when moving from noise category 30–35 dB to 35–40 dB, where the prevalence increased from 1% to 10%, and this continued to increase to 13.7% at 40–46 dB [11].

In this study, the proportion of subjects being annoyed (or highly annoyed), both outdoors and indoors, in majority cases decreased significantly with a greater distance from the nearest wind turbine (Figure 4). However, contrary to the majority of other investigations, when analyzing annoyance (both outdoors and indoors) in various noise categories, no significant differences were noted. Observed was only a tendency to an increasing percentage of subjects being highly annoyed outdoors or annoyed (and highly annoyed) indoors along with increasing noise levels (Figure 4). It is worth noting that generally the relationships between the distance (or noise) and “high annoyance” were more pronounced than in the case of “annoyance”.

Nevertheless, the results of our study are partly in line with the observations from earlier cross-sectional investigations, since the results of logistic regression have shown that odds ratio of being highly annoyed indoors increased approximately 1.1 times per each 1 dB increase of sound pressure level and noise level itself explains approximately 2% of variance in high annoyance indoors (Table 11).

Such relationships were not found when analyzing the high annoyance outdoors. However, about 1% of variance in high annoyance can be explained by the distance itself, and odds ratio that respondents would be highly annoyed outdoors decreased approximately three times if the distance increased by 1 km (OR = 0.33; 95% CI: 0.12–0.92) (Table 10).

In this study over half of the subjects were classified as sensitive to noise (68.3%) and landscape littering (63.6%). Of the respondents, 40.3% and 32.3% declared negative (“very negative” or “negative”) attitude towards wind turbines in general and their visual impact in particular, respectively. The majority of subjective (individual) factors (i.e., sensitivity to various environmental nuisances, attitude to wind turbines, physical and mental health status) were correlated to each other (Table 3). In particular, there was a relatively high positive correlation between the attitude toward wind turbines in general and the attitude to their visual impact in particular as well as between the respondents’ sensitivity to noise and sensitivity to landscape littering. Furthermore, a percentage of subjects negatively assessing wind turbines in general and their impact on landscape in particular, decreased significantly when moving from noise category >45 dB (or 40–45 dB) to <35 dB. On the other hand, the greater distance from the nearest wind turbine, the smaller the percentage of subjects with negative opinion towards wind turbines (Table 2).

To analyze the influence of subjective (or individual) and objective (situational) factors (including noise level and distance) on annoyance rating binary logistic regression was applied. However, due to high positive correlations between the aforesaid subjective variables in the study subjects, only some of them, namely the general attitude towards wind turbines and sensitivity to noise and landscape littering were taken into consideration. Other analyzed factors, having a possible impact on annoyance perception, were age, gender, education, terrain shape, road traffic intensity, time since the start-up of the wind farm, power and height of the nearest wind turbine, economic benefits from the wind farm and having wind turbines visible from dwelling.

It has been shown that the probability of being highly annoyed outdoors was significantly associated with the sound pressure level (or distance), attitude towards wind turbines, sensitivity to noise as well as terrain shape. On the other hand, the annoyance perception indoors was correlated with the noise level (or distance), age, attitude towards wind turbines, sensitivity to noise and traffic intensity. No significant relationships were observed in case of the remaining factors (Table 8 and Table 9). 

Almost all study subjects could see one or more wind turbines from their dwelling, while only a few (6.1%) of them had profits from the wind turbines. Thus, it is not surprising that no significant associations were noted between these factors and noise annoyance.

When exploring the factors affecting a high annoyance outdoors using binary logistic regression, the highest value of the explained variance (approximately 66%) was obtained for the models containing a distance or noise level (only in case of high annoyance) and general attitude to wind turbines, noise sensitivity and terrain shape. However, a similar percentage of explained variance (approximately 61%) was obtained when only the noise level (or distance) and the general attitude to wind turbines were included into the model.

On the other hand, when testing high annoyance indoors, the greatest values of explained variance (approximately 48%) were obtained for the model containing the noise level and general attitude to wind turbines, noise sensitivity and road-traffic intensity. Moreover, likewise in case of a probability of being annoyed outdoors, the percentage of explained variance decreased slightly (to approximately 45%) if only the noise level (or distance) and general attitude to wind turbines were taken into consideration.

Generally, the odds ratio of being highly annoyed by the wind turbine noise (both outdoors and indoors) increased with increasing noise level, higher sensitivity to noise and more negative attitude towards wind turbines, while it decreased with an increasing distance from the nearest wind turbine. Similarly, hilly or mountainous (vs. flat) terrain and no or low-intensity (vs. increased intensity) road-traffic increased the risk of being annoyed outdoors and indoors, respectively. Thus, the findings obtained in this study confirmed the conclusions from the earlier Dutch studies, that annoyance perception was associated with terrain and urbanization, i.e., a rural area increased the risk of perception and annoyance in comparison with a suburban area, in a rural setting a diversified (hilly or rocky) terrain increased the risk compared with a flat terrain [3].

It is worth emphasizing that inclusion of the road-traffic intensity into the model is equivalent to taking into account the prevalence of masking noise, namely road-traffic noise. Furthermore, our findings are also in line with Scandinavian observations that people living with other background noises are less affected by the wind turbine noise than those from the quiet areas [5]. However, it has been shown that the road traffic noise can provide a significant masking of the wind farm noise, but only at intermediate levels of the wind turbine sound (35–40 dBA), not at higher or lower levels [38].

Over one-fifth of our study subjects assessed their physical health negatively, while nearly every fifth or fourth subject frequently reported stress symptoms such as headache (21.3%), undue tiredness (27.1%), feeling nervous, tense or stressed (28.0%). Furthermore, of total respondents, 25.1% had often difficulty falling asleep, while 19.3% of them complained of insomnia. Nearly half (45.8%) of them often woke up not well-rested. Regarding sleep disturbances, 27.3% of the subjects stated they were disturbed in their sleep by noise from various sources (including the road traffic noise and wind turbine noise) when sleeping with an open window, while 15.1% of them were disturbed when sleeping with a closed window. Moreover, about one-third (33.7%) of the study subjects stated that they were wakened by the wind turbines.

Respondents examined using the GHQ-12 obtained a mean score at the level of 12.5 ± 6.1 which was similar to the normative result for the reference Polish population (11.17 ± 5.11). Furthermore, the prevalence of “cases” (35%) in the study population was comparable to that observed in the Polish healthy working population (27%) [18].

As to various health and well-being aspects, there were no significant differences between noise or distance categories (Figure 6). However, people being annoyed (or highly) annoyed by the wind turbine noise more frequently than those not annoyed assessed their mental health negatively, reported stress symptoms, and stated that they were disturbed in their sleep by the noise from wind turbines. Furthermore, a greater percentage of the so-called “cases” according to the GHQ-12 score were recognized among annoyed (or highly annoyed) subjects. Those findings were supported by the binary logistic regression which confirmed that almost all variables measuring various health and well-being aspects were positively associated with a high annoyance related to the wind turbine noise both outdoors and indoors, while there were no significant associations with the noise level or the distance from the nearest wind turbine.

The above mentioned findings confirmed some conclusions from earlier Scandinavian cross-sectional studies, since analysis of the combined data from these investigations showed that the subjects who were annoyed by the wind turbine noise were more likely to report sleep disturbances, feeling tense, stressed and irritable [38]. Our findings are also in agreement with the results from CNHS which did not support an association between the exposure to the wind turbine noise (up to 46 dBA) and self-reported health effects (e.g., migraines, tinnitus, dizziness), sleep disturbances and sleep disorders, as well as elevated self-reported and objectively defined measures of stress [12,13]. On the other hand, our results are contrary to the findings obtained by Nissenbaum et al. [30] indicating that the subjects living within 1.4 km of an industrial wind turbine could not sleep well, they were more sleepy during the day, and had worse SF36 Mental Components Scores, as compared to those living further than 1.4 km.

Results of binary logistic regression were the basis for determination of exposure-response relationships expressed as the probability (percentage) of being highly annoyed in the function of the calculated A-weighted sound pressure level (or distance) and other explanatory variables, including the general attitude to wind turbines, sensitivity to noise, and road-traffic intensity or the terrain shape. According to the aforesaid curves, namely those relating annoyance to sound pressure level (or distance) and attitude towards turbines, in areas where the A-weighted sound pressure level exceeds 40 dB, above 2% and 52% of the people with positive and negative attitudes to the wind farm, respectively, might be highly annoyed outdoors (Figure 9b). At such noise exposures, above 27% of the subjects with negative opinions about wind farms might assess the wind turbine noise as annoying indoors (Figure 10b). On the other hand, at the distance of 1000 m the wind turbine noise might be perceived as highly annoying outdoors by approximately 2% and 43% of the people with a positive and negative attitude towards wind farms, respectively (Figure 9a). At the same distance, up to 21% of the subjects (with negative attitude to wind farms) might be highly annoyed by the wind turbine noise indoors (Figure 10a).

According to the obtained exposure-response curves we can estimate that even among the people with very negative attitudes to the wind turbines, at relatively long (>2.1 km) distances from the wind turbines the percentage of those evaluating the noise as very tiresome (both outdoors and indoors) will not exceed 10%.

The relationships between the self-reported annoyance and distance (and other factors) seem to be useful in selecting setback distances to reduce or avoid potential noise complaints from, or potential effects to, people living close to wind farms. This is important, since several unreliabilities related to the calculations might result in over- or underestimation of noise levels experienced in everyday life in the vicinity of wind farms.

It is worth emphasizing that minimum setback distances have been established world-wide to reduce or avoid potential noise complaints from, or potential effects to, the people living close to wind turbines. For example, in Ontario a minimum setback distance of 550 m must exist between the center of the base of the wind turbine and the nearest noise receptor (e.g., a building or campground). That minimum setback distance was developed through noise modeling under worst-case conditions to give a conservative estimate of the distance at which the noise attains the A-weighted sound level of 40 dB [4], the noise level that corresponds to the WHO night-noise guideline, a health-based limit [39]. On the other hand, the Queensland guidelines specify a minimum setback distance of 1.5 km. According to Davy et al. [40], this came from a National Health and Medical Research Council information paper which states: “Although individuals may perceive aspects of wind farm noise at greater distances, it is unlikely that the wind farm noise will be considered disturbing at distances of >1500 m”.

According to exposure-relationship curves which were determined in this study, at such a distance less than 10% of people with negative attitude towards wind turbines might be highly annoyed indoors. However, to keep the percentage of people highly annoyed when outdoors in the value of less than 10% the distance should be greater than 2.1 km.

Taking into account the fact that evaluation of the wind turbines noise annoyance depends on many interrelated subjective and objective factors and that the interrelations between those factors are not known sufficiently as yet, it would be advisable to create the noise annoyance model involving only objective (or situational) factors such as e.g., the distance of dwellings from the nearest wind turbine.

## 5. Conclusions

It has been shown that the wind turbine noise at the calculated A-weighted sound pressure level of 33–50 dB was perceived as annoying or highly annoying by 46% and 28% of respondents living within 204–1726 m from the nearest wind turbine, respectively. On the other hand, 34% and 18% of them said that they were annoyed or highly annoyed indoors, respectively.

The perception of annoyance and high annoyance was associated with the A-weighted sound pressure level or the distance from the nearest wind turbine, general attitude to wind farms, noise sensitivity and terrain shape (annoyance outdoors) or road-traffic intensity (annoyance indoors). About 48–66% of variance in annoyance might be explained by the aforesaid factors. However, the relationships between the distance (or sound pressure level) and “high annoyance” were more pronounced than those with “annoyance”. 

The odds ratio of being highly annoyed by the wind turbine noise (both outdoors and indoors) increased with an increasing noise level, higher sensitivity to noise and more negative attitude towards wind turbines, while it decreased with an increasing distance from the nearest wind turbine. Similarly, hilly or mountainous (vs. flat) terrain and no or low-intensity (vs. increased intensity) road-traffic increased the risk of being annoyed outdoors and indoors, respectively.

The percentage of respondents being annoyed by the wind turbine noise strongly depended on their attitude to wind turbines. It was estimated that in areas where the A-weighted sound pressure level exceeds 40 dB, above 52% of people with negative attitude to wind farms might be highly annoyed outdoors, as opposed to 2% of those with a positive attitude. On the other hand, at a distance of 1000 m the wind turbine noise might be perceived as highly annoying outdoors by approximately 43% and 2% of people with negative and positive attitude to wind turbines, respectively.

There was no significant association between the noise level (or distance) and various health and well-being aspects. However, almost all variables measuring health and well-being aspects, including stress symptoms and self-reported sleep disturbances, were positively associated with high annoyance related to the wind turbine noise both outdoors and indoors.

The relationships between annoyance and distance (and other factors) seem to be useful in selecting setback distances to reduce or avoid potential noise complaints from, or potential effects to, people living close to wind farms. This is important, since several unreliabilities related to the calculations might result in over- or underestimation of noise levels experienced in everyday life in the vicinity of wind farms.

Nevertheless, further studies are needed, in particular those using both self-reported and objective measures, before firm conclusions can be drawn on the impact of the wind turbine noise on health and well-being.

## Figures and Tables

**Table 1:** Characteristics of investigated areas.

No.	Area	Localization	Number of Respondents	Wind Farm	Power Installed	Wind Turbines			
						Quantity Pcs.	Power	Sound Power Level	Type
1	32.2 km^2^	Kuyavian-Pomeranian Province, Lipno County	64	Farm No. 1	34 MW	17	2 MW	104.0 dB	Vestas V-90
					600 kW	6	100 kW	99.0 dB	-
2	45.2 km^2^	West Pomeranian Province, Koszalin County	52	Farm No. 2	50 MW	25	2 MW	105.5 dB	Vestas V-80
3	73.9 km^2^	West Pomeranian Province, Bialogard County	21	Farm No. 3	90 MW	60	1.5 MW	105.1 dB	Fuhrländer Fl 1500 77
4	4.7 km^2^	West Pomeranian Province, Slawno County	35	Farm No. 4	20 MW	10	2 MW	105.5 dB	Vestas V-80
					660 kW	5	132 kW	99.0 dB	Seewind
5	4.9 km^2^	West Pomeranian Province, Slawno County	35	Farm No. 5	22.5 MW	9	2.5 MW	103.5 dB	Nordex N90
6	15.6 km^2^	West Pomeranian Province, Slawno County	28	Farm No. 6	50 MW	20	2.5 MW	105.0 dB	GE 2.5xl
7	11.2 km^2^	West Pomeranian Province, Puck County	102	Farm No. 7	8 MW	4	2 MW	105.5 dB	Vestas V80
					10 MW	4	2.5 MW	103.5 dB	Nordex N80
					3.2 MW	4	800 kW	102.5 dB	Enercon E40
					1.2 MW	2	600 kW	99.0 dB	WestWind
				Farm No. 8	22 MW	11	2 MW	105.0 dB	Gamesa G87 T78
8	20.87 km^2^	Podlasie Province, Suwalki County	106	Farm No. 9	41.4 MW	18	2.3 MW	107.0 dB	Siemens SWT-2.3-93
9	14.55 km^2^	Subcarpathia Province, Krosno County	74	Farm No. 10	36 MW	18	2 MW	104.2 dB	-

**Table 2:** Study group characteristics.

Characteristic	Respondents						
	Total	Noise Category			Distance Category		
		≤40 dB	40–45 dB	> 45 dB	≤400 m	400–800 m	>800 m
Age (year) (M ± SD)	46.7 ± 15.8	48.5 ± 15.9	46.5 ± 15.7	44.4 ± 15.6	44.2 ± 15.1	47.3 ± 15.8	45.8 ± 15.8
Range	18.0–88.4	19.0–88.4	18.2–83.3	18.0–78.7	18.0–78.2	19.4–88.4	18.2–82.4
Gender: male (%)	41.8	38.2	41.5	47.4	40.8	44.1	35.5
Occupation (%)							
employed	53.8	44.4 ^a^	55.2	62.5 ^a^	69.4 ^b^	53.5	48.4 ^b^
students	1.7	1.5	1.7	2.1	4.1	1.4	1.6
pensioners	23.4	27.1	22.9	19.8	16.3	24.6	23.0
farmers	15.1	11.3	15.6	18.8	24.5	15.0	11.5
Residence: detached houses/farms (%)	84.8	84.5	82.7	91.6	83.7	86.4	80.8
Sensitive to (%)							
noise	68.3	70.3	65.2	74.5	85.4 ^a^	65.3 ^a^	69.7
landscape littering	63.6	66.4	60.1	70.2	77.1	63.4	58.8
air pollution	65.9	76.2 ^a^	59.7 ^a^	70.2	83.3 ^a^	61.9 ^a^	70.0
Negative (%)							
Attitude to wind turbines in general	40.3	38.3 ^a^	38.6 ^b^	47.3 ^a^^,b^	48.9 ^a^	43.2^b^	28.0 ^a^^,b^
Attitude to visual impact of wind turbines	32.3	34.4	29.9	36.8	36.7	35.1^a^	22.7 ^a^
Self-assessment of physical health	23.0	22.7	22.3	25.3	28.6	23.4	19.7
Self-assessment of hearing status	15.2	13.0	17.1	12.9	11.9	17.7	9.6
Score in the GHQ-12 (M ± SD)	12.5 ± 6.1	12.3 ± 5.6	11.5 ± 4.8	12.0 ± 7.6	13.3 ± 8.2	12.7 ± 6.1	11.5 ± 4.8
Range	1.0–36.0	1.0–31.0	2.0–36.0	2.0–33.0	3.0–33.0	1.0–36.0	5.0–31.0
Respondents (%)							
Classified as cases on the basis of the GHQ-12 score	35.0	32.5	37.4	32.0	31.7	38.3	28.3
Who can see at least one wind turbine from their dwelling	93.4	91.0	94.1	94.8	98.0	93.9	90.2
With profits from wind turbines	6.1	0.0 ^a^	6.6 ^b^	12.6 ^a^^,b^	6.1	7.9 ^a^	0.9 ^a^
Study group (n)	517	133	288	96	49	346	122

M—mean; SD—standard deviation; GHQ-12—12-item Goldberg’s General Health Questionnaire; ^a,b^ Significant differences between noise or distance categories (*p* < 0.01667).

**Table 3:** Relationships between various subjective variables in the study group ^1^. Statistically significant correlations are presented in bold numbers (*p* < 0.0013989).

	Spearman’s Rank Correlation Coefficient								
	1	2	3	4	5	6	7	8	9
1. Age		**−0.470**	0.050	−0.032	0.012	0.106	0.093	**−0.413**	0.096
2. Education			0.152	0.176	−0.014	−0.101	0.016	**0.285**	−0.040
3. Sensitivity to noise				**0.674**	**−0.432**	**0.342**	**0.306**	**−0.244**	**0.229**
4. Sensitivity to landscape littering					**−0.508**	**0.464**	**0.438**	**−0.222**	**0.318**
5. Opinion about wind turbines impact on health						**−0.756**	**−0.661**	**0.422**	**−0.345**
6. Attitude to wind turbines in general							**0.757**	**−0.424**	**0.467**
7. Attitude to visual impact of wind turbines								**−0.260**	**0.337**
8. Self-assessment of physical health									**−0.406**
9. Score in the GHQ-12									

^1^ Except for age and the GHQ-12 score, the analyzed variables are presented on 5-point or 6-point rating scale. To avoid the risk of mass significance, *p* < 0.001389 was required for statistical significance; GHQ-12—12-item Goldberg’s General Health Questionnaire.

**Table 4:** Summary results of noise calculations for areas where the respondents lived, with a distance of their dwellings from the nearest wind turbine.

Study Group	Number of Subjects	A-Weighted Sound Pressure Level (dB)	Day-Evening-Night Noise Level, L_den_ (dB)	Distance (m)
		M ± SD (Me)		
		Range		
Total subjects	517	42.1 ± 3.1 (41.7)	46.8 ± 3.1 (46.4)	665 ± 194 (656)
		33.7–49.9	38.4–54.6	204–1726
Noise category				
≤40 dB	133	38.1 ± 1.4 (38.1)	42.8 ± 1.4 (42.8)	819 ± 163 (795)
		33.7–40.0	38.4–44.7	353–1726
40–45 dB	288	42.4 ± 1.5 (42.1)	47.1 ± 1.5 (46.8)	669 ± 156 (648)
		40.1–46.0	44.8–50.7	235–998
>45 dB	96	46.4 ± 1.5 (46.3)	51.1 ± 1.5 (51.0)	440 ± 97 (441)
		43.6–49.9	48.3–54.6	204–617
Distance category				
≤400 m	49	46.3 ± 2.4 (46.7)	51.0 ± 2.4 (51.4)	344 ± 51 (365)
		39.3–49.9	44.0–54.6	204–92
400–800 m	346	42.4 ± 2.7 (42.7)	47.1 ± 2.7 (47.4)	622 ± 108 (618)
		37.0–47.6	41.7–52.3	403–800
>800	122	39.4 ± 2.2 (40.4)	44.1 ± 2.2 (45.1)	915 ± 125 (891)
		33.7–42.0	38.4–46.7	801–1726

M—mean; SD—standard deviation; Me—median; CI—confidence interval.

**Table 5:** Comparison of proportions (with 95% confidence intervals) of respondents who were annoyed (or highly annoyed) by the wind turbine noise and other environmental nuisances ^1^.

Environmental Nuisances	Proportion of Respondents (%)			
	95% CI			
	Annoyed		Highly Annoyed	
	Outdoors	Indoors	Outdoors	Indoors
Wind turbine noise	46.4 (42.2–50.7)	33.7 (29.7–37.8)	28.0 (24.4–32.1)	18.4 (15.3–22.0)
Road-traffic noise	28.4 (24.7–32.5)	18.4 (15.3–22.0)	12.0 (9.5–15.1)	9.7 (7.4–12.6)
Noise from hand held and stationary power tools	17.0 (14.0–20.5)	10.8 (8.4–13.8)	6.6 (4.7–9.1)	3.9 (2.5–5.9)
Noise from agricultural machinery	12.8 (10.2–15.9)	7.7 (5.7–10.4)	4.3 (2.8–6.4)	2.5 (1.4–4.3)
Odors from agriculture	18.6 (15.5–22.2)	6.4 (4.6–8.9)	7.4 (5.4–10.0)	2.7 (1.6–4.6)
Aircraft noise	12.8 (10.2–15.9)	7.2 (5.2–9.7)	3.9 (2.5–5.9)	2.9 (1.7–4.8)
Odors from industry	18.2 (15.1–21.8)	7.0 (5.1–9.5)	8.9 (6.7–11.7)	3.7 (2.3–5.7)
Railway noise	0.8 (0.2–2.1)	1.0 (0.4–2.3)	0.6 (0.1–1.8)	0.2 (0.0–1.2)

^1^ To avoid the risk of mass significance, *p* < 0.00625 was required for statistical significance. There were significant differences between proportions of respondents who were annoyed (or highly annoyed) by the wind turbine noise and other environmental nuisances (*p* < 0.00625); CI−confidence interval.

**Table 6:** Proportions (with 95% confidence intervals) of respondents who noticed and were annoyed or highly annoyed by the wind turbine noise in total and in each of the noise and distance categories ^1^.

Group of Respondents	Proportion of Respondents (%)					
	(95% Cl)					
	Perceived Wind Turbine Noise		Annoyed by Wind Turbine Noise		Highly Annoyed by Wind Turbine Noise	
	Outdoors	Indoors	Outdoors	Indoors	Outdoors	Indoors
Total	65.2	45.1	46.4	33.7	28.0	18.4
	(61.0–69.2)	(40.8–49.4)	(42.2–50.7)	(29.7–37.8)	(24.4–32.1)	(15.3–22.0)
Noise category						
≤40 dB	67.7	42.9	47.4	28.6	24.1	14.3
	(59.3–75.0)	(34.8–51.4)	(39.1–55.8)	(21.6–36.8)	(17.6–32.1)	(9.3–21.4)
40–45 dB	63.2	44.1	44.4	33.3	27.1	18.8
	(57.5–68.6)	(38.5–49.9)	(38.8–50.2)	(28.1–39.0)	(22.3–32.5)	(14.7–23.7)
>45 dB	67.7	51.0	51.0	41.7	36.5	22.9
	(57.8–76.2)	(41.2–60.8)	(41.2–60.8)	(32.3–51.7)	(27.5–46.5)	(15.6–32.4)
Distance category						
≤400 m	77.6	59.2	53.1	44.9	36.7	24.5
	(63.9–87.1)	(45.2–71.8)	(39.4–66.3) ^a^	(31.9–58.7) ^a^	(24.7–50.8) ^a^	(14.5–38.3) ^a^
400–800 m	66.8	50.3	50.3	37.9	30.6	21.1
	(61.6–71.5)	(45.0–55.5)	(45.0–55.5)	(32.9–43.1) ^b^	(26.0–35.7) ^b^	(17.1–25.7) ^b^
>800 m	55.7	24.6	32.8	17.2	17.2	8.2
	(46.9–64.2)	(17.8–33.0)	(25.1–41.6) ^a^	(11.5–25.0) ^a,b^	(11.5–25.0) ^a,b^	(4.4–14.6) ^a,b^

^1^ Individual noise and distance categories were compared in pairs. To avoid the risk of mass significance, *p* < 0.0167 was required for statistical significance; ^a,b^ Significant differences between distance categories (*p* < 0.0167); CI—confidence interval.

**Table 7:** Proportions of respondents (with 95% confidence intervals) who assessed their physical health negatively, being categorized as “cases” based on the GHQ-12 score, and frequently reported various complaints in total and in subgroups of subjects annoyed and not annoyed by the wind turbine noise ^1^.

	Proportion of Respondents (%)				
	95% CI				
	Total	Not Annoyed Outdoors	Annoyed Outdoors	Not Highly Annoyed Outdoors	Highly Annoyed Outdoors
Negative self-assessment of physical health	23.0	11.1	36.6	14.2	23.0
	(19.5–26.8)	(7.8–15.4)	(30.7–42.9)	(11.0–18.2)	(19.5–26.8)
Negative self-assessment of hearing status	15.2	6.3	24.3	8.2	15.2
	(12.1–19.1)	(3.7–10.7)	(18.9–30.7)	(5.5–12.1)	(12.1–19.1)
Cases according to the GHQ-12 score	35.0	19.9	52.6	23.8	35.0
	(30.6–39.8)	(15.2–25.7)	(45.6–59.6)	(19.3–29.0)	(30.6–39.8)
Headache	21.3	11.2	32.9	14.5	21.3
	(18.0–25.0)	(8.0–15.5)	(27.3–39.1)	(11.3–18.5)	(18.0–25.0)
Dizziness	13.0	5.8	21.3	7.0	13.0
	(10.3–16.2)	(3.5–9.3)	(16.5–26.9)	(4.8–10.1)	(10.3–16.2)
Heartache	13.0	5.8	21.3	7.8	13.0
	(10.3–16.2)	(3.5–9.3)	(16.5–26.9)	(5.5–11.0)	(10.3–16.2)
Fatigue (undue tiredness)	27.1	14.1	42.1	17.5	27.1
	(23.4–31.1)	(10.5–18.7)	(36.0–48.4)	(14.0–21.7)	(23.4–31.1)
Insomnia	19.3	8.3	32.1	9.4	19.3
	(16.2–23.0)	(5.6–12.2)	(26.5–38.2)	(6.8–12.9)	(16.2–23.0)
Pain and stiffness in the back, neck or shoulders	24.2	16.2	33.3	20.2	24.2
	(20.7–28.1)	(12.4–21.1)	(27.7–39.5)	(16.4–24.6)	(20.7–28.1)
Pulsation in ears	8.7	1.8	16.7	3.8	8.7
	(6.6–11.5)	(0.7–4.3)	(12.5–21.9)	(2.2–6.3)	(6.6–11.5)
Dyspnea	9.5	6.9	12.5	6.2	9.5
	(7.2–12.3)	(4.4–10.5)	(8.9–17.4)	(4.1–9.2)	(7.2–12.3)
Nervousness, tension or stress	28.0	16.2	41.7	18.5	28.0
	(24.4–32.1)	(12.4–21.1)	(35.6–48.0)	(14.9–22.8)	(24.4–32.1)
Tinnitus	8.3	2.5	15.0	3.5	8.3
	(6.2–11.0)	(1.1–5.3)	(11.0–20.1)	(2.0–6.0)	(6.2–11.0)
Waking up well rested	54.2	63.2	43.8	60.2	54.2
	(49.8–58.4)	(57.3–68.6)	(37.6–50.1)	(55.2–65.1)	(49.8–58.4)
Having difficulty in falling asleep	25.1	13.4	38.8	15.9	25.1
	(21.6–29.1)	(9.8–17.9)	(32.8–45.1)	(12.5–19.9)	(21.6–29.1)
Awakened by noise with closed window	27.3	12.6	44.2	14.8	27.3
	(23.6–31.3)	(9.2–17.1)	(38.0–50.5)	(11.5–18.8)	(23.6–31.3)
Awakened by noise with open window	15.1	5.8	25.8	6.2	15.1
	(12.3–18.5)	(3.5–9.3)	(20.7–31.7)	(4.1–9.2)	(12.3–18.5)
Awakened by wind turbines	33.7	1.7	62.5	9.5	33.7
	(29.0–38.7)	(0.4–5.3)	(55.5–69.0)	(6.4–14.0)	(29.0–38.7)

^1^ There were statistically significant differences between subgroups of respondents being annoyed (or highly annoyed) and not annoyed (or not highly annoyed), both outdoors and indoors, by the wind turbine noise (*p* < 0.05); GHQ-12—12-item Goldberg’s General Health Questionnaire; CI—confidence interval.

**Table 8:** Association between perception outdoors of high annoyance related to the wind turbine noise (dependent, binary variable) and the calculated A-weighted sound pressure level (or distance) as well as subjective (individual) and objective factors (independent variables) tested by logistic regression.

Explanatory Variables	High Annoyance Outdoors							
	Odds Ratio (95% CI)	*p*	Odds Ratio (95% CI)	*p*	Odds Ratio (95% CI)	*p*	Odds Ratio (95% CI)	*p*
Age	1.01 (0.98–1.03)	0.530	1.01 (0.98–1.03)	0.610	1.01 (0.99–1.03)	0.461	1.01 (0.98–1.03)	0.534
Gender	1.46 (0.77–2.80)	0.248	1.48 (0.78–2.83)	0.234	1.41 (0.74–2.68)	0.290	1.43 (0.75–2.71)	0.273
Education	0.93 (0.68–1.28)	0.669	0.93 (0.67–1.28)	0.650	0.93 (0.68–1.29)	0.677	0.93 (0.68–1.28)	0.671
Sensitivity to noise	1.72 (1.19–2.47)	0.004	1.74 (1.21–2.52)	0.003	-	-	-	-
Sensitivity to landscape littering	-	-	-	-	1.32 (0.93–1.86)	0.121	1.32 (0.93–1.87)	0.116
Attitude to wind turbines	5.02 (3.50–7.21)	0.000	4.99 (3.48–7.16)	0.000	4.91 (3.37–7.14)	0.000	4.88 (3.36–7.09)	0.000
Sound pressure level	1.15 (1.03–1.29)	0.014	-	-	1.15 (1.02–1.28)	0.017	-	-
Distance from the nearest wind turbine	-	-	0.13 (0.02–0.75)	0.023	-	-	0.16 (0.03–0.90)	0.038
Power of the nearest wind turbine	0.65 (0.18–2.30)	0.503	0.58 (0.16–2.11)	0.411	0.74 (0.22–2.52)	0.626	0.67 (0.19–2.33)	0.526
Height of the nearest wind turbine	1.02 (0.98–1.06)	0.283	1.04 (1.00–1.08)	0.077	1.02 (0.98–1.06)	0.370	1.03 (0.99–1.08)	0.123
Duration of wind farm operation	0.86 (0.69–1.07)	0.181	0.95 (0.78–1.15)	0.573	0.88 (0.71–1.10)	0.260	0.97 (0.80–1.17)	0.720
Having turbines visible from the dwelling	0.34 (0.08–1.48)	0.150	0.39 (0.09–1.65)	0.199	0.43 (0.10–1.84)	0.255	0.49 (0.12–2.04)	0.325
Profits from wind turbines	1.34 (0.32–5.71)	0.688	1.46 (0.34–6.16)	0.609	1.17 (0.28–4.94)	0.833	1.28 (0.30–5.38)	0.740
Terrain shape	2.74 (1.22–6.16)	0.015	2.53 (1.14–5.60)	0.022	3.02 (1.36–6.71)	0.007	2.81 (1.28–6.15)	0.010
Road-traffic intensity	1.43 (0.44–4.70)	0.554	1.81 (0.59–5.58)	0.302	1.52 (0.48–4.83)	0.479	1.87 (0.62–5.62)	0.262

**Table 9:** Association between perception indoors of high annoyance related to the wind turbine noise (dependent, binary variable) and the calculated A-weighted sound pressure level (or distance) as well as subjective (individual) and objective factors (independent variables) tested by logistic regression.

Explanatory Variables	High Annoyance Indoors							
	Odds Ratio (95% CI)	*p*	Odds Ratio (95% CI)	*p*	Odds Ratio (95% CI)	*p*	Odds Ratio (95% CI)	*p*
Age	1.03 (1.00–1.05)	0.026	1.02 (1.00–1.05)	0.031	1.03 (1.00–1.05)	0.023	1.02 (1.00–1.05)	0.028
Gender	1.14(0.62–2.12)	0.672	1.15 (0.62–2.13)	0.653	1.12 (0.61–2.07)	0.715	1.13 (0.61–2.09)	0.688
Education	1.23 (0.90–1.68)	0.203	1.23 (0.89–1.68)	0.206	1.20 (0.87–1.65)	0.265	1.20 (0.88–1.66)	0.253
Sensitivity to noise	1.50 (1.03–2.17)	0.033	1.51 (1.04–2.19)	0.032	-	-	-	-
Sensitivity to landscape littering	-	-	-	-	1.34 (0.94–1.92)	0.103	1.32 (0.93–1.89)	0.121
Attitude to wind turbines	3.43 (2.44–4.82)	0.000	3.39 (2.41–4.76)	0.000	3.38 (2.37–4.82)	0.000	3.37 (2.36–4.80)	0.000
Sound pressure level	1.12 (1.01–1.24)	0.040	-	-	1.11 (1.00–1.24)	0.043	-	-
Distance from the nearest wind turbine	-	-	0.22 (0.04–1.22)	0.083	-	-	0.25 (0.05–1.38)	0.112
Power of the nearest wind turbine	1.11 (0.33–3.72)	0.867	0.99 (0.27–3.70)	0.989	1.18 (0.35–3.96)	0.784	1.07 (0.32–3.61)	0.917
Height of the nearest wind turbine	1.01 (0.97–1.05)	0.679	1.02 (0.98–1.06)	0.351	1.01 (0.97–1.05)	0.768	1.02 (0.98–1.06)	0.411
Duration of wind farm operation	0.97 (0.82–1.16)	0.750	1.03 (0.89–1.21)	0.668	0.99 (0.83–1.18)	0.917	1.05 (0.90–1.23)	0.513
Having turbines visible from the dwelling	0.75 (0.15–3.74)	0.725	0.80 (0.16–3.88)	0.781	0.82 (0.17–4.04)	0.810	0.89 (0.19–4.20)	0.878
Profits from wind turbines	0.67 (0.29–1.55)	0.354	0.68(0.30–1.56)	0.361	0.75 (0.33–1.71)	0.492	0.76 (0.33–1.71)	0.503
Terrain shape	0.99 (0.21–4.63)	0.986	1.03(0.22–4.79)	0.965	0.96 (0.21–4.37)	0.954	1.01 (0.22–4.66)	0.990
Road-traffic intensity	3.65 (1.06–12.61)	0.04	3.96(1.18–13.32)	0.026	3.81 (1.11–13.09)	0.033	4.11 (1.23–13.76)	0.022

**Table 10:** Factors affecting the noise annoyance perception outdoors—results of logistic regression analyses ^1^.

Explanatory Variable	Model No.	Odds Ratio (95% CI)	*p*	Pseudo-R^2^	CCR
		High Annoyance Outdoors			
Distance	1	0.11 (0.02–0.52)	0.006	0.659	87.7
Traffic intensity		4.23 (2.25–7.97)	0.000		
Attitude to wind turbines		5.45 (3.88–7.64)	0.000		
Sensitivity to noise		1.56 (1.11–2.21)	0.012		
Distance	2	0.11 (0.02–0.51)	0.005	0.645	87.5
Attitude to wind turbines		4.43 (2.40–8.19)	0.000		
Sensitivity to noise		6.09 (4.37–8.48)	0.000		
Distance	3	0.18 (0.04–0.78)	0.022	0.625	87.3
Traffic intensity		5.94 (4.24–8.34)	0.000		
Attitude to wind turbines		1.69 (1.21–2.36)	0.002		
Distance	4	0.18 (0.04–0.75)	0.019	0.606	87.3
Attitude to wind turbines		6.77 (4.85–9.44)	0.000		
Distance	5	0.28 (0.09–0.83)	0.021	0.214	75.4
Sensitivity to noise		2.63 (2.02–3.43)	0.000		
Distance	6	0.29 (0.10–0.86)	0.027	0.174	72.7
Traffic intensity		5.34 (3.40–8.39)	0.000		
Distance	7	0.33 (0.12–0.92)	0.034	0.013	72.0
Sound pressure level	8	1.14 (1.03–1.25)	0.008	0.658	87.5
Traffic intensity		4.08 (2.19–7.60)	0.000		
Attitude to wind turbines		5.62 (3.98–7.92)	0.000		
Sensitivity to noise		1.54 (1.09–2.17)	0.014		
Sound pressure level	9	1.15 (1.05–1.27)	0.003	0.646	88.1
Attitude to wind turbines		4.31 (2.35–7.90)	0.000		
Sensitivity to noise		6.32 (4.50–8.87)	0.000		
Sound pressure level	10	1.11 (1.02–1.22)	0.023	0.625	86.3
Traffic intensity		6.01 (4.28–8.44)	0.000		
Attitude to wind turbines		1.68 (1.21–2.34)	0.002		
Sound pressure level	11	1.13 (1.03–1.23)	0.010	0.608	86.5
Attitude to wind turbines		6.87 (4.91–9.60)	0.000		
Sound pressure level	12	0.28 (0.09–0.83)	0.021	0.214	75.4
Sensitivity to noise		2.63 (2.02–3.43)	0.000		
Sound pressure level	13	1.06 (1.00–1.13)	0.071	0.170	72.0
Traffic intensity		5.29 (3.37–8.29)	0.000		
Sound pressure level	14	1.09 (1.01–1.17)	0.071	0.009	72.0

^1^ Various models were created with the sound pressure level (or distance) and attitude to wind turbines, noise sensitivity and terrain shape as possible explanatory variables; Pseudo-R^2^—the Nagelkerke coefficient of determination, i.e., a measure of explained variance [25]; CCR—correct classification rate; CI—confidence interval.

**Table 11:** Factors affecting noise annoyance perception indoors—results of logistic regression analyses ^1^.

Explanatory Variable	Model No.	Odds Ratio (95% CI)	*p*	Pseudo-R^2^	CCR
		High Annoyance Indoors			
Distance	1	0.29 (0.06–1.29)	0.103	0.476	86.7
Traffic intensity		3.43 (1.33–8.84)	0.011		
Attitude to wind turbines		3.40 (2.51–4.61)	0.000		
Sensitivity to noise		1.51 (1.05–2.16)	0.024		
Distance	2	0.21 (0.05–0.93)	0.040	0.458	86.5
Attitude to wind turbines		3.73 (2.76–5.05)	0.000		
Sensitivity to noise		1.47 (1.04–2.08)	0.028		
Distance	3	0.27 (0.06–1.20)	0.085	0.463	87.1
Traffic intensity		3.21 (1.26–8.13)	0.014		
Attitude to wind turbines		3.90 (2.90–5.24)	0.000		
Distance	4	0.20 (0.04–0.90)	0.036	0.446	87.3
Attitude to wind turbines		4.27 (3.18–5.71)	0.000		
Distance	5	0.22 (0.06–0.76)	0.017	0.168	69.9
Sensitivity to noise		2.50 (1.83–3.40)	0.000		
Distance	6	0.34 (0.10–1.18)	0.089	0.125	81.6
Traffic intensity		7.59 (3.23–17.85)	0.000		
Distance	7	0.23 (0.07–0.77)	0.017	0.019	81.6
Sound pressure level	8	1.10 (1.00–1.20)	0.039	0.480	86.7
Traffic intensity		3.72 (1.45–9.52)	0.006		
Attitude to wind turbines		3.41 (2.52–4.61)	0.000		
Sensitivity to noise		1.50 (1.05–2.14)	0.027		
Sound pressure level	9	1.10 (1.01–1.20)	0.033	0.458	63.4
Attitude to wind turbines		3.76 (2.78–5.09)	0.000		
Sensitivity to noise		1.46 (1.04–2.05)	0.031		
Sound pressure level	10	1.11 (1.01–1.21)	0.025	0.468	87.1
Traffic intensity		3.52 (1.40–8.87)	0.008		
Attitude to wind turbines		3.89 (2.90–5.22)	0.000		
Sound pressure level	11	1.11 (1.01–1.21)	0.024	0.448	87.5
Attitude to wind turbines		4.29 (3.20–5.75)	0.000		
Sound pressure level	12	1.08 (1.01–1.17)	0.031	0.164	81.0
Sensitivity to noise		2.48 (1.82–3.37)	0.000		
Sound pressure level	13	1.08 (1.01–1.16)	0.029	0.130	87.1
Traffic intensity		7.96 (3.39–18.70)	0.000		
Sound pressure level	14	1.09 (1.01–1.17)	0.022	0.017	81.6

^1^ Various models were created with the sound pressure level (or distance) and attitude to wind turbines, noise sensitivity and road-traffic intensity as possible explanatory variables; Pseudo-R^2^—the Nagelkerke coefficient of determination, i.e., a measure of explained variance [25]; CCR—correct classification rate. CI—confidence interval.

**Table 12:** Estimated coefficients for various logistic models of high annoyance due to the wind turbine noise outdoors.

	**Constant**	**SE**	***p***	**Distance**	**SE**	***p***	**Terrain Shape**	**SE**	***p***	**Attitude to Wind Turbines**	**SE**	***p***	**Sensitivity to Noise**	**SE**	***p***
1	−5.494	0.768	0.000	−2.218	0.796	0.006	1.443	0.322	0.000	1.695	0.172	0.000	0.446	0.176	0.012
2	−4.581	0.650	0.000	−2.212	0.781	0.005	1.489	0.313	0.000	1.806	0.168	0.000	−	−	−
3	−5.409	0.758	0.000	−1.717	0.748	0.022	−	−	−	1.782	0.172	0.000	0.525	0.170	0.002
4	−4.290	0.632	0.000	−1.725	0.732	0.019	−	−	−	1.912	0.170	0.000	−	−	−
5	−1.148	0.402	0.004	−1.244	0.559	0.027	1.676	0.230	0.000	−	−	−	−	−	−
6	−2.788	0.540	0.000	−1.277	0.554	0.022	−	−	−	−	−	−	0.968	0.135	0.000
7	−0.208	0.354	0.556	−1.119	0.525	0.034	−	−	−	−	−	−	−	−	−
	**Constant**	**SE**	***p***	**Sound Pressure Level**	**SE**	***p***	**Terrain Shape**	**SE**	***p***	**Attitude to Wind Turbines**	**SE**	***p***	**Sensitivity Noise**	**SE**	***p***
8	−12.368	2.258	0.000	0.129	0.048	0.008	1.405	0.317	0.000	1.726	0.175	0.000	0.431	0.174	0.014
9	−12.046	2.200	0.000	0.141	0.048	0.003	1.461	0.308	0.000	1.844	0.172	0.000	−	−	−
10	−11.067	2.141	0.000	0.107	0.047	0.023	−	−	−	1.794	0.173	0.000	0.519	0.168	0.002
11	−10.496	2.066	0.000	0.120	0.046	0.010	−	−	−	1.927	0.171	0.000	−	−	−
12	−6.112	1.480	0.000	0.059	0.033	0.075	−	−	−	−	−	−	0.961	0.134	0.000
13	−4.375	1.356	0.001	0.057	0.032	0.071	1.666	0.229	0.000	−	−	−	−	−	−
14	−3.354	1.338	0.013	0.057	0.032	0.071	−	−	−	−	−	−	−	−	−

SE—standard error.

**Table 13:** Estimated coefficients for various logistic models of high annoyance due to the wind turbine noise indoors.

**Model No.**	**Constant**	**SE**	***p***	**Distance**	**SE**	***p***	**Traffic Intensity**	**SE**	***p***	**Attitude to Wind Turbines**	**SE**	***p***	**Sensitivity to Noise**	**SE**	***p***
1	−5.904	0.911	0.000	−1.247	0.764	0.103	1.231	0.482	0.011	1.224	0.154	0.000	0.411	0.182	0.024
2	−4.846	0.770	0.000	−1.556	0.757	0.040	−	−	−	1.316	0.154	0.000	0.386	0.176	0.028
3	−4.994	0.785	0.000	−1.328	0.769	0.085	1.165	0.474	0.014	1.361	0.150	0.000	−	−	−
4	−4.076	0.670	0.000	−1.613	0.766	0.036	−	−	−	1.451	0.149	0.000	−	−	−
5	−3.111	0.636	0.000	−1.519	0.636	0.017	−	−	−	−	−	−	0.915	0.158	0.000
6	−2.472	0.592	0.000	−1.065	0.625	0.089	2.027	0.435	0.000	−	−	−	−	−	−
7	−0.528	0.408	0.196	−1.486	0.620	0.017	−	−	−	−	−	−	−	−	−
	**Constant**	**SE**	***p***	**Sound Pressure Level**	**SE**	***p***	**Traffic Intensity**	**SE**	***p***	**Attitude to Wind Turbines**	**SE**	***p***	**Sensitivity to Noise**	**SE**	***p***
8	−10.701	2.059	0.000	0.093	0.045	0.039	1.314	0.478	0.006	1.225	0.154	0.000	0.403	0.182	0.027
9	−9.903	2.013	0.000	0.096	0.045	0.033	−	−	−	1.325	0.154	0.000	0.378	0.174	0.031
10	−10.170	2.009	0.000	0.101	0.045	0.025	1.258	0.470	0.008	1.359	0.150	0.000	−	−	−
11	−9.415	1.967	0.000	0.101	0.045	0.024	−	−	−	1.457	0.149	0.000	−	−	−
12	−7.502	1.682	0.000	0.081	0.038	0.031	−	−	−	−	−	−	0.908	0.157	0.000
13	−6.533	1.584	0.000	0.079	0.036	0.029	2.075	0.434	0.000	−	−	−	−	−	−
14	−5.075	1.574	0.001	0.085	0.037	0.022	−	−	−	−	−	−	−	−	−

SE—standard error.

**Table 14:** Association between various aspects of health and well-being (binary dependent variables) and distance or noise level (independent continuous variable) or annoyance due to the wind turbine noise outdoors (or indoors) (independent binary variable) tested by logistic regression ^1^.

	Explanatory Variable					
	Odds Ratio (95% CI)					
	Distance ^a^	Highly Annoyed Outdoors ^b^	Highly Annoyed Indoors ^b^	A–weighted Sound Pressure Level ^a^	Highly Annoyed Outdoors ^c^	Highly Annoyed Indoors ^c^
Negative self–assessment of physical health	0.51 (0.16–1.67)	**5.48 (3.38–8.88)**	**7.05 (4.15–12.01)**	1.05 (0.98–1.13)	**5.46 (3.37–8.83)**	**6.99 (4.11–11.87)**
Negative self–assessment of hearing status	0.63 (0.13–3.06)	**4.77 (2.61–8.73)**	**5.71 (3.03–10.75)**	1.06 (0.97–1.16)	**4.64 (2.55–8.47)**	**5.46 (2.92–10.24)**
“Cases” according to the GHQ–12 score	0.58 (0.20–1.64)	**5.36 (3.34–8.59)**	**4.68 (2.75–7.98)**	1.04 (0.98–1.11)	**5.30 (3.31–8.49)**	**4.62 (2.72–7.85)**
Headache	0.68 (0.22–2.11)	**3.99 (2.50–6.36)**	**4.07 (2.44–6.77)**	1.00 (0.93–1.07)	**4.04 (2.53–6.43)**	**4.16 (2.50–6.93)**
Dizziness	0.27 (0.06–1.11)	**5.31 (3.04–9.29)**	**5.99 (3.38–10.63)**	1.05 (0.97–1.14)	**5.41 (3.10–9.45)**	**6.12 (3.45–10.85)**
Heartache	0.68 (0.17–2.83)	**4.36 (2.48–7.65)**	**4.47 (2.48–8.05)**	0.98 (0.90–1.07)	**4.45 (2.54–7.80)**	**4.64 (2.58–8.37)**
Fatigue (undue tiredness)	0.86 (0.30–2.47)	**5.36 (3.42–8.39)**	**4.33 (2.65–7.09)**	0.98 (0.92–1.04)	**5.46 (3.48–8.55)**	**4.48 (2.73–7.34)**
Insomnia	0.37 (0.11–1.26)	**8.37 (5.02–13.95)**	**5.90 (3.50–9.94)**	1.03 (0.96–1.11)	**8.51 (5.11–14.18)**	**6.05 (3.58–10.20)**
Pain and stiffness in back. neck or shoulders	0.57 (0.19–1.75)	**1.97 (1.25–3.08)**	**2.07 (1.25–3.43)**	1.01 (0.95–1.08)	**1.99 (1.27–3.12)**	**2.11 (1.27–3.49)**
Pulsation in ears	0.86 (0.16–4.73)	**6.57 (3.30–13.08)**	**5.42 (2.77–10.61)**	1.01 (0.91–1.11)	**6.51 (3.28–12.93)**	**5.40 (2.76–10.54)**
Dyspnea	0.89 (0.17–4.81)	**3.13 (1.66–5.93)**	**4.78 (2.46–9.30)**	0.97 (0.88–1.07)	**3.19 (1.69–6.03)**	**4.95 (2.54–9.64)**
Nervousness. tension or stressed	0.48 (0.17–1.35)	**5.00 (3.23–7.74)**	**4.20 (2.59–6.81)**	1.01 (0.95–1.07)	**5.12 (3.31–7.92)**	**4.35 (2.68–7.05)**
Tinnitus	0.59 (0.10–3.49)	**6.68 (3.28–13.59)**	**5.94 (2.97–11.85)**	1.00 (0.91–1.11)	**6.76 (3.33–13.74)**	**6.08 (3.05–12.13)**
Respondent woke up well–rested	0.79 (0.32–1.96)	**0.42 (0.28–0.62)**	**0.50 (0.32–0.80)**	1.01 (0.95–1.06)	**0.42 (0.28–0.63)**	**0.51 (0.32–0.81)**
Difficulty in falling asleep	0.85 (0.29–2.48)	**5.59 (3.55–8.80)**	**5.26 (3.20–8.65)**	1.04 (0.97–1.11)	**5.47 (3.48–8.60)**	**5.13 (3.13–8.42)**
Awakened by noise with open window	1.08 (0.39–2.99)	**8.38 (5.35–13.12)**	**8.13 (4.93–13.39)**	1.01 (0.95–1.07)	**8.20 (5.26–12.79)**	**7.99 (4.85–13.14)**
Awakened by noise with closed window	0.94 (0.25–3.49)	**9.00 (5.18–15.62)**	**7.76 (4.49–13.40)**	1.02 (0.95–1.11)	**8.78 (5.07–15.19)**	**7.55 (4.38–13.01)**
Awakened by wind turbines	0.94 (0.25–3.49)	**38.03 (20.36–71.02)**	**19.50 (10.10–37.64)**	1.03 (0.96–1.11)	**38.17 (20.44–71.28)**	**19.87 (10.27–38.42)**

^1^ Statistically significant relationships are presented in bold numbers (*p* < 0.05); ^a^ Adjusted for age and gender; ^b^ Adjusted for age, gender and distance from the nearest wind turbine; ^c^ Adjusted for age, gender and the calculated A-weighted sound pressure level.
